# Surgical site infection following surgery for hand trauma: a systematic review and meta-analysis

**DOI:** 10.1177/17531934231193336

**Published:** 2023-08-22

**Authors:** Justin C. Wormald, Alexander J. Baldwin, Hayat Nadama, Abigail Shaw, Ryckie G. Wade, Dani Prieto-Alhambra, Jonathan A. Cook, Jeremy N. Rodrigues, Matthew L. Costa

**Affiliations:** 1 Kadoorie Centre for Critical Care Research and Education, Nuffield Department of Orthopaedics, Rheumatology and Musculoskeletal Sciences (NDORMS), University of Oxford, Oxford, UK; 2 Department of Burns and Plastic Surgery, Stoke Mandeville Hospital, Aylesbury, UK; 3 Department of Cardiothoracic Surgery, Liverpool Heart and Chest Hospital, Liverpool, UK; 4 Department of Plastic Surgery, Salisbury NHS Foundation Trust, Salisbury District Hospital, Odstock, Salisbury, UK; 5 Leeds Institute for Medical Research, University of Leeds, Leeds, UK; 6 Botnar Institute for Musculoskeletal Sciences, Nuffield Department of Orthopaedics, Rheumatology and Musculoskeletal Sciences (NDORMS), University of Oxford, Oxford, UK; 7 Warwick Clinical Trials Unit, Warwick Medical School, University of Warwick, Coventry and Department of Plastic Surgery, Stoke Mandeville Hospital, Buckinghamshire Healthcare NHS Trust, Aylesbury, UK

**Keywords:** Hand surgery, trauma, infection, systematic review

## Abstract

Surgical site infection is the most common healthcare-associated infection. Surgical site infection after surgery for hand trauma is associated with increased antibiotic prescribing, re-operation, hospital readmission and delayed rehabilitation, and in severe cases may lead to amputation. As the risk of surgical site infection after surgery for hand trauma remains unclear, we performed a systematic review and meta-analysis of all primary studies of hand trauma surgery, including randomized controlled trials, cohort studies, case-control studies and case series. A total of 8836 abstracts were screened, and 201 full studies with 315,618 patients included. The meta-analysis showed a 10% risk of surgical site infection in randomized control trials, with an overall risk of 5% when all studies were included. These summary statistics can be used clinically for informed consent and shared decision making, and for power calculations for future clinical trials of antimicrobial interventions in hand trauma.

## Introduction

Surgical site infection (SSI) is the most common healthcare-associated infection (World Health Organization, 2018), with an estimated risk of 3–5% after all surgery in the United Kingdom (UK) (National Institute for Health and Care Excellence, 2019). SSIs lead to increased morbidity and mortality beyond the original indication for surgery and are potentially preventable. Existing SSI statistics cited in guidelines are often based on large observational studies in general surgical populations (Gibbons et al., 2011; Leaper et al., 2008). Data on the risk of SSI in hand surgery, particularly trauma, are sparse. SSI after hand surgery can lead to additional interventions, including further surgery and delayed rehabilitation (Menendez et al., 2015). This leads to impaired functional recovery, which is critical in a predominantly young and working population (De Putter et al., 2012). Prevention and cost-effective management of SSI following hand surgery is essential in terms of antibiotic regulation and optimizing outcomes; there are over 250,000 hand trauma operations per year in the UK alone (Warwick, 2018). The importance of assessing the risk of SSI in limb trauma has been highlighted as a top priority in the Complex Fractures Priority Setting Partnership (Complex Fractures PSP | James Lind Alliance, 2022). This is particularly important in hand trauma, where approximately 50% of injuries are fractures (Manley et al., 2019).

Without knowing the baseline risk of SSI for hand trauma surgery, it is difficult to educate patients honestly and fully about the advantages and disadvantages of different treatment options, especially where non-operative interventions are an option (Giddins, 2015). Furthermore, it is impossible to prioritize research on SSI in hand trauma surgery if the scale of the problem is unknown (James Lind Alliance Priority Setting Partnership: Common Conditions Affecting the Hand and Wrist, 2017). The purpose of this systematic review is to produce summary statistics and data that can potentially be used as information in clinical care and research.

## Methods

We performed a systematic review in accordance with the Preferred Reporting Items for Systematic Reviews and Meta-Analyses (PRISMA) statement (Higgins and Green, 2011;  Moher et al., 2009). The *a priori* protocol was registered on the PROSPERO database (CRD42020215825).

### Search strategy

We used relevant search terms to create a text term strategy and a database-specific Medical Subject Headings (MeSH) term strategy for each database (Appendix S1). No date or language restrictions were applied to the database searches, including Medline, EMBASE and CINAHL (via the NICE Healthcare Databases Advanced Search interface), the Cochrane Central Register of Controlled Trials (CENTRAL) and clinicaltrials.gov from database inception to January 2021 (extended to July 2022). Reference lists of included articles were screened for further relevant publications. In parallel, we manually searched the grey literature using Google Scholar.

### Study eligibility

Inclusion and exclusion criteria according to the PICO framework were as follows – Population: adult participants (≥18 years old) with hand and/or wrist injuries; Intervention/comparator: any surgical intervention, with or without a control arm; Outcomes: reported SSI by any classification system or diagnostic method. Studies with mixed adult and paediatric populations were included if the data were reported separately for each population. We excluded case reports, opinion pieces, systematic reviews and meta-analyses, narrative and scoping reviews, and anatomical, cadaveric, laboratory and biomechanical studies. Cohort studies were defined by the inclusion criteria of the study, for example, if the inclusion criteria were based on a population characteristic such as distal radial fracture. Case series were defined as studies that included participants based on the intervention, for example, patients who underwent external fixation of a distal radial fracture.

### Data extraction

Four authors independently screened the abstracts using a pre-specified checklist of the inclusion criteria (Appendix S2). Data were extracted by four authors using a predefined electronic form. The accuracy of data extraction was crossed-checked within the group of authors. The unit of analysis was the patient, not the hand/wrist (Wade et al., 2016).

### Data analysis

We performed a descriptive analysis to summarize the elements of the included study characteristics. Meta-analysis of proportions was performed to determine pooled risk of SSI across study populations (Wang, 2018). This method allows generation of pooled risk statistics that are weighted according to the size of the individual study populations, giving more weight to larger studies and less to smaller studies. Subgroup analyses were performed for methodological (study design, study type, infection classification) and clinical subgroups (injury site, injury type, intervention). Statistical heterogeneity was assessed using the chi-squared test (*p* < 0.10 = statistically significant heterogeneity) and the *I*^2^ measure (Higgins et al., 2003). Mixed-effects meta-regression was used to explore heterogeneity in the clinical and methodological variables of interest. The following categorical fixed effects were used for univariable models in the first instance: study design, study type, infection definition, the risk of methodological bias, injury site, injury type and intervention. To explore whether the method of fixation was independently associated with the risk of SSI, a multivariable meta-regression model was setup including all the above factors (Online Appendix S4). ‘Study design’ was removed from the multivariable model as it was strongly co-linear with study type. Odds ratios (OR) with 95% confidence intervals (CI) were generated.

### Risk of bias

We used the Cochrane Risk of Bias 2 tool (RoB 2) to assess RoB in randomized control trials (RCTs) and quasi-RCTs (Sterne et al., 2019). RCTs were then classified as having a low, moderate or high risk of bias. The risk of bias for cohort studies, case-control studies and case series was assessed as good, fair or poor using the National Institute of Health (NIH) National Heart, Lung and Blood Institute Study Assessment Tools for the respective study designs (NIH National Heart Lung and BIood Institute, 2018). To be consistent with the Cochrane RoB 2 tool, we converted this rating to low, moderate or high risk of bias.

## Results

**Figure 1.**
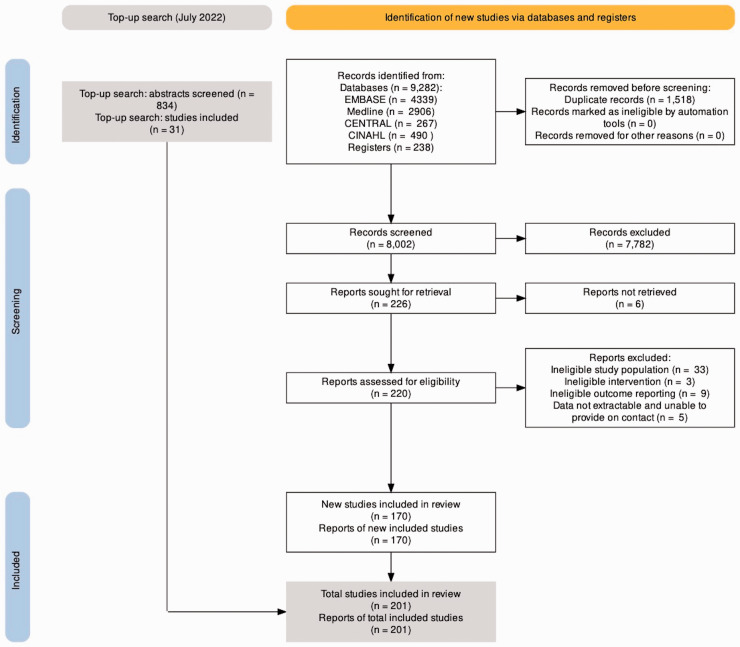
PRISMA flow chart.

Four reviewers (JCRW, AJB, HN, and AS) independently screened 8002 abstracts against predefined eligibility criteria to determine potential for inclusion (Figure 1). The reviewers then evaluated 220 full-text articles in detail for eligibility. Google Translate was used for non-English languages to determine eligibility. After review, 170 studies were eligible for inclusion. An additional top-up search in July 2022 resulted in a further 31 included studies, for a total of 201 included studies.

### Characteristics of included studies

There were 315,618 study participants in all included studies and 3131 individual cases of SSI (Table 1, Online Tables S1–4, Online Appendix S5). The individual proportion of cases that developed an SSI in each study ranged from 0% to 47%. The included studies were published between 1973 and 2022, and originated from Europe, America, Asia, Africa and Australia. The study populations ranged from three participants to 132,650 participants. Between 1973 and 2022 there was no detectable change in the incidence of SSI in the literature (Online Figure S1).

**Table 1.** Characteristics of included studies.

Study design	*n* studies	*n* total	SSI risk	95% CI lower	95% CI upper	Risk of bias (% studies)		
RCT	27	3,867	10.1	9.0	11.4	15	22	63
Cohort	79	306,244	1.5	1.5	1.6	33	31	48
Case control	2	37	NA	NA	NA	100		
Case series	93	5264	8.6	7.7	9.6	17	48	34
Overall	201	315,618	5.0	3.9	6.1	23	33	44

RCT: randomized control trial; *n* total: total participants in study design population; SSI: surgical site infection; 95% CI: confidence interval.

Of the 201 included studies, 132 were retrospective observational studies, 42 were prospective observational studies and 27 were RCTs. Fourteen studies used a recognized classification or definition of SSI. Most studies evaluated hand injuries (105, 52%). External fixation was evaluated in 21 studies, Kirschner wire fixation in 43, open reduction and internal fixation (ORIF) in 53, soft-tissue reconstruction in 37 and 47 studies evaluated multiple interventions (Online Appendix S6).

### Risk of bias

**Figure 2.**
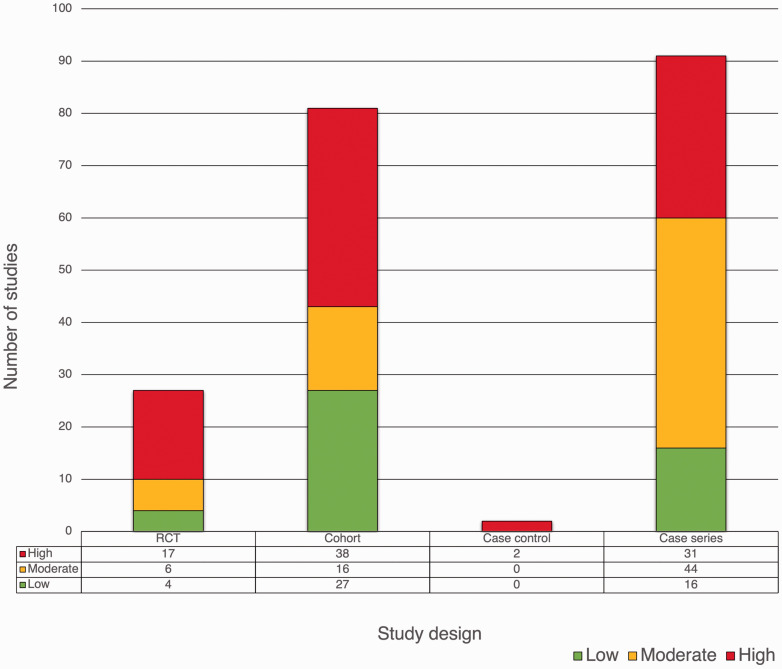
Risk of bias of included studies.

Eighty-eight studies had a high risk of bias (Figure 2). Of the 27 included RCTs, four had a low risk of bias. Of the 81 cohort studies included, 27 were at low risk of bias (33% of cohorts). The two case-control studies were at high risk of bias. Of the 91 case series included, 16 were at low risk of bias.

### Meta-analysis of the risk of SSIs

All studies were eligible for inclusion in the meta-analysis. The summary risk of SSI across all studies was 5.0% [CI 3.9 to 6.1] (Table 2 and Online Figure S2). There was statistical heterogeneity as indicated by high *I*^2^ statistics. Random-effects analyses handled the heterogeneity better than common (fixed) effects and so only the former are discussed.

**Table 2.** Meta-analysis of SSI risk in hand trauma.

	No. of studies	No. of patients	Pooled risk (%)		Meta-regression			
			SSI risk	95% CI	OR	95% CI	*P*-value	
Methodological variables								
Study design								
RCT	27	3867	10.1	9.0–11.4	0.3	0.1–1.8	0.19	^a^
Cohort	79	306,224	1.5	1.5–1.6	0.2	0.0–1.1	0.06	^a^
Case series	93	5264	8.6	7.7–9.6	0.3	0.0–1.5	0.23	^a^
Case control	2	37	21.8	11.3–38.0	0.3^d^	0.0–1.5	0.12	^a^
Study type								
Retrospective	129	307,857	4.7	3.6–6.0	0.8	0.5–1.3	0.41	^b^
Prospective	70	7555	6.0	4.2–8.3	0.4^d^	0.1–2.7	0.35	^b^
Infection definition								
Unknown	45	8128	4.4	2.7–7.2	1.9	0.8–4.3	0.12	^b^
Clinical	142	264,420	5.3	4.1–6.9	1.9	0.9–3.7	0.07	^b^
Definition	14	42,864	4.1	1.9–8.8	0.4^d^	0.1–2.7	0.35	^b^
Risk of Bias								
Low	47	37,841	4.1	2.4–6.8	0.9	0.5–1.6	0.81	^b^
Moderate	66	271,453	4.4	3.1–6.3	0.8	0.5–1.2	0.31	^b^
High	88	6118	6.1	4.4–8.3	0.4^d^	0.1–2.7	0.35	^b^
Clinical variables								
Injury site								
Wrist	81	299,805	4.5	3.3–6.2	0.7	0.4–1.1	0.15	^b^
Mixed	15	3806	5.6	2.9–10.7	0.8	0.4–1.6	0.52	^b^
Hand	105	11,801	5.5	4.1–7.2	0.4^d^	0.1–2.7	0.35	^b^
Injury type^SD^								
Open	56	7005	4.9	2.6–9.3	1.3	0.3–1.1	0.41	^b^
Closed	90	154,564	4.2	2.7–6.4	0.4^d^	0.1–2.7	0.35	^b^
Intervention^SD^								
STR	37	2460	4.6	2.8–8.5	0.3	0.1–0.8	0.02^c^	^b^
ORIF	53	169,098	2.0	1.2–3.1	0.2	0.1–0.3	<0.001	^b^
K-wire	43	5739	7.0	4.7–10.4	0.5	0.3–1.1	0.11	^b^
Ex-Fix	21	1127	15.4	7.7–28.6	0.4^d^	0.1–2.7	0.35	^b^
Overall	201	315,618	5.0	3.9–6.1				

a Unadjusted; heterogenity: *I*^2^ 96%, *p* = <0.0001.

b Adjusted, heterogenity: *I*^2^ 90%, *p* = <0.0001 SD mixed study populations excluded from analyses.

c Not significant with mixed study populations excluded .

d Intercept.

SSI: surgical site infection; RCT: randomized controlled trial; STR: soft tissue reconstruction; ORIF: open reduction internal fixation; K-wire: Kirschner wire; Ex-Fix: external fixation; OR: odds ratio; CI: confidence interval.

### Subgroup analyses by methodology (Online Figure S3)

The risk of SSI in the RCT study population (*n* = 3867) was substantially higher than in the overall analysis above: 10% [CI 9.0 to 11.4] (Online Figure S4.). The risk of SSI in the case series population (*n* = 5412) was also higher than the overall summary statistic: 8.6% [CI 7.7 to 9.6]. The cohort study population (*n* = 306,136) had an extremely low summary risk of SSI: 1.5% [CI 1.5 to 1.6]. The two case-control studies (*n* = 37) were small and had a high risk of bias and were excluded from the study design analysis. Univariate meta-regression showed that the study design did not account for the observed heterogeneity in the data set (*p = *0.06 to 0.23).

Subgroup analysis by study type (retrospective versus prospective) revealed no difference in the risk of SSI in prospective study populations: 6% [CI 4.2 to 8.3], compared with retrospective studies: 4.7% [CI 3.6 to 6.0]; multivariate meta-regression (*p = *0.41) (Online Figure S5). The definition of infection did not affect the risk of SSI in the study populations. When an established definition of infection was used, the risk of SSI was 4.1% [CI 1.9 to 8.8]. When a ‘clinical’ definition was used, the risk was 5.3% [CI 4.1 to 6.9]; and when no definition was described, the risk was 4.4% [CI 2.7 to 7.2] (Online Figure S6). The risk of bias also did not influence the risk of SSI. The risk of SSI was 4.1% [CI 2.4 to 6.8] in studies with low risk of bias, 4.4% [CI 3.1 to 6.3] in studies with moderate risk and 6.1% [CI 4.4 to 8.3] in studies with high risk (Online Figure S7).

### Subgroup analysis by clinical factors (Online Figure S8)

We removed the mixed population studies for the injury type (open versus closed) and intervention subgroup analysis to ensure the different risk statistics were an accurate representation of the populations. Anatomical site and type of injury did not statistically appear to affect SSI risk (Online Figure S9, Online Figure S10). SSI risk varied according to type of intervention. The SSI risk following soft-tissue reconstruction was 4.6% [CI 2.8 to 8.5]. For participants who underwent fracture fixation, the SSI risk for ORIF was 2.0% [CI 1.2 to 3.1], for Kirschner wire fixation was 7.0% [CI 4.7 to 10.4] and for external fixation was 15% [CI 7.7 to 28.6] (Online Figure S11). After controlling for other factors using multivariate meta-regression, ORIF had significantly lower risk of SSI (OR 0.2, *p = *<0.001 [CI 0.1 to 0.3]) compared with external fixation and the lowest SSI risk of all surgical interventions.

## Discussion

This systematic review is a comprehensive overview of studies of surgical interventions for hand trauma that reported postoperative infection as an outcome, synthesizing data from 315,618 patients. Using meta-analysis, we have generated summary statistics that can be used to infer the risk of SSI for both clinical and research purposes. The overall risk of SSI for hand trauma surgery is approximately 5%, at least as high as the UK NICE estimate of 3–5%, and probably higher as it is generally accepted that national SSI statistics are underestimated if SSIs occurring outside the hospital are missed (Gibbons et al., 2011; Leaper et al., 2008).

We found a higher risk of SSI in RCT populations, which is mirrored in RCTs in other surgical specialities. In a meta-analysis of 21 RCTs evaluating antimicrobial sutures in abdominal wounds, the pooled risk of SSI was over 10% in both the intervention and control groups (De Jonge et al., 2017). The ‘true’ risk of SSI in hand trauma surgery is likely to be between 5% (overall risk) and 10% (risk in RCTs), based on our current understanding of SSI research. SSIs in hand surgery are poorly documented in the medical records and may be missed in studies that rely on retrospective case review (Seigerman et al., 2020). It is unlikely to be less than 5% despite the low SSI risk seen in the subgroup analysis of the cohort study. This subgroup analysis is biased by very large, retrospective registry studies, which are unreliable for detecting SSI because they rely solely on valid recording and coding (Leaper et al., 2013; Smyth and Emmerson, 2000). Looking at the largest included studies, the SSI risk ranges from 0.1% to 2.3% in studies with over 10,000 participants (Constantine et al., 2022; Galivanche et al., 2021; Mahmood et al., 2022; Wei et al., 2021). These four studies are all on distal radial fracture fixation, mostly ORIF, and all are retrospective. These studies carry considerable weight in the meta-analysis due to their large study populations and will therefore influence the overall risk of SSI, but particularly the risk in the study design and intervention subgroup analyses.

Other than study design, aspects of research design and quality did not appear to result in substantially different risks of SSI. The use of a predefined infection classification system (centers for disease control and prevention (CDC), most commonly) did not change the risk of SSI: 4.2% compared with 4.4% when no definition was given (Online Figure S9). Similarly, studies with a high risk of bias had only a slightly higher risk of SSI than those with a moderate or low risk of bias (6% compared with 4%). One explanation for the highest risk of SSI in RCTs could be that they are all prospective, have lower attrition and use predefined criteria, which reduces measurement bias and leads to more reliable data. We explored this in our meta-regression to determine the effect of study type (retrospective versus prospective) on study design in terms of SSI risk. We found that the prospective nature of a study does not explain the higher risk of SSI in RCTs, and therefore other variables associated with clinical trials must account for the increased risk, such as improved overall study quality (Appendix S3).

The risk of SSI for those undergoing Kirschner wire fixation of hand and wrist fractures was 7%, which is consistent with existing meta-analyses (Wormald et al., 2017a, 2017b, 2019). The risk of SSI for injuries requiring soft-tissue reconstruction was 5%, which is consistent with the overall risk in this dataset and the literature (Baldwin et al., 2021; Davies et al., 2020; Platt and Page, 1995).

We did not observe a higher risk of infection for open injuries compared with closed injuries, as has traditionally been assumed. One theory is that because of the perceived increased risk of infection (drawn from the non-upper limb literature), open injuries may be debrided more quickly and receive adjuvant antimicrobial interventions (Bachoura et al., 2011; Thakore et al., 2015). In contrast, closed injuries, which are thought to have a lower baseline risk of SSI, may receive less antimicrobial prophylaxis. It was not surprising that the risk of SSI was slightly higher for hand injuries compared with wrist injuries, as the latter had a substantially larger number of ORIF studies, which have an independently lower risk of SSI.

There is evidence that the risk of SSI is higher in developing countries, with pooled risks as high as 12% overall, and ranging from 7.6 per 100 clean surgical procedures to 39.2 per 100 dirty surgical procedures (Allegranzi et al., 2011). In the recently published FALCON trial, based in 54 developing countries and involving 5788 participants, the overall risk of SSI was 22%, rising to 30% for dirty wounds (Ademuyiwa et al., 2021). It is likely that the risk of SSI following hand trauma would be similarly increased in developing countries and should be a future priority for global health research in hand trauma.

The heterogeneity of the included studies was a limitation, but we explored potential causes using subgroup analysis and meta-regression with cautious interpretation. Our comprehensive search strategy, updated in July 2022, may have missed some studies. Funnel plot analysis (Online Figure S12) showed no clear evidence of publication bias.

The risk of SSI in hand trauma surgery may be as high as 10% according to RCT data, with an overall risk of 5% when all studies are included in the analysis. This provides new, definitive summary statistics that can be used clinically for informed consent and shared decision making and to inform power calculations for future clinical studies of antimicrobial interventions in hand trauma surgery.

## Supplemental Material

sj-pdf-1-jhs-10.1177_17531934231193336 - Supplemental material for Surgical site infection following surgery for hand trauma: a systematic review and meta-analysis Click here for additional data file. Supplemental material, sj-pdf-1-jhs-10.1177_17531934231193336 for Surgical site infection following surgery for hand trauma: a systematic review and meta-analysis by Justin C. Wormald Alexander J. Baldwin, Hayat Nadama, Abigail Shaw, Ryckie G. Wade, Dani Prieto-Alhambra, Jonathan A. Cook, Jeremy N. Rodrigues and Matthew L. Costa in Journal of Hand Surgery (European Volume)
