# Supplementary material for: Surgical site infection following surgery for hand trauma: a systematic review and meta-analysis
Source: J Hand Surg Eur Vol. 2023 Aug 22;48(10):998–1005. doi: 10.1177/17531934231193336 (PMC10616993; doi:10.1177/17531934231193336)
Supplement: sj-pdf-1-jhs-10.1177_17531934231193336 - Supplemental material for Surgical site infection following surgery for hand trauma: a systematic review and meta-analysis [file sj-pdf-1-jhs-10.1177_17531934231193336.pdf]

Surgical site infection following surgery for hand trauma A systematic review and meta-analysis

Wormald JCR, Baldwin AJ, Nadama H, Shaw A, Wade RG, Prieto-Alhambra D, Cook JA, Rodrigues JN, Costa ML

Contents

Online Figure 1. SSI incidence over time

Online Figure 2. Meta-analysis – all included studies

Online Figure 3. Meta-analysis – methodological subgroup analyses

Online Figure 4. Meta-analysis – subgroup analysis by study design

Online Figure 5. Meta-analysis – subgroup analysis by study type

Online Figure 6. Meta-analysis – subgroup analysis by infection definition

Online Figure 7. Meta-analysis – subgroup analysis by risk of bias

Online Figure 8. Meta-analysis – clinical subgroup analyses

Online Figure 9. Meta-analysis – subgroup analysis by injury site

Online Figure 10. Meta-analysis – subgroup analysis by injury type

Online Figure 11. Meta-analysis – subgroup analysis by intervention

Online Figure 12. Meta-analysis – funnel plot

Appendix 1. Search Strategies

Appendix 2 - Abstract Decision Tree

Appendix 3. Meta-regression output

Appendix 4. SRMA R Script

Appendix 5. Full dataset

Appendix 6. References for included studies

Online Table 1 - Characteristics of RCTs

Online Table 2 - Characteristics of Cohort Studies

Online Table 3 - Characteristics of Case Control Studies

Online Table 4 - Characteristics of Case Series

Supplementary figure 1. SSI incidence over time

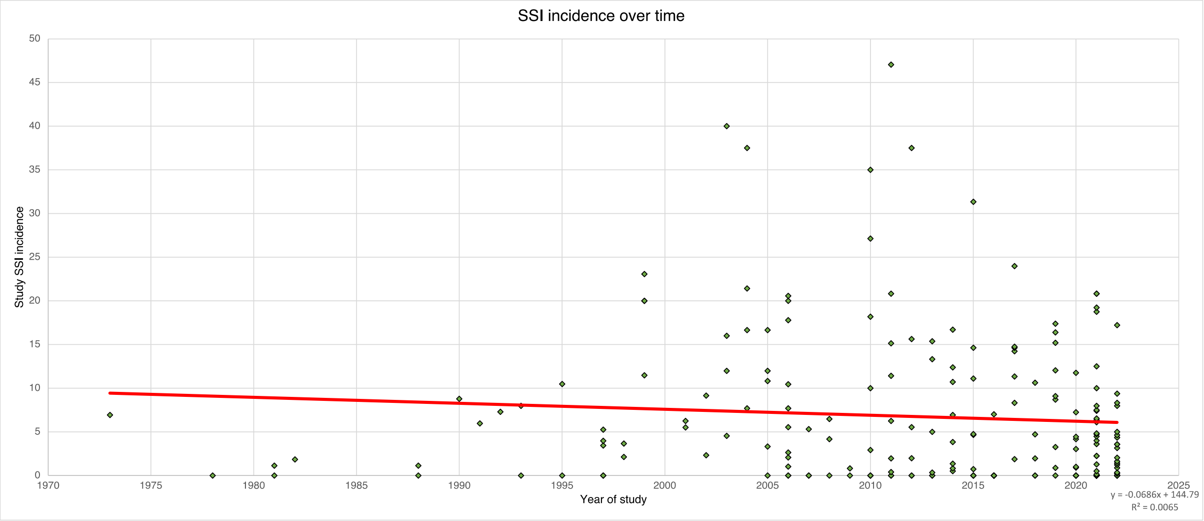

Supplementary Figure 2. Meta-analysis – all included studies

Study	Cases	Total	Risk of SSI	95% C.I.
Abouelela 2020	1	33	3.03	[0.43; 18.61]
Abulsoud 2021	0	23	0.00	[0.13; 25.94]
Adkison 1982	1	54	1.85	[0.26; 12.01]
Afshar 2020	0	21	0.00	[0.14; 27.74]
Aigner 2014	6	721	0.83	[0.37; 1.84]
Akmaz 2003	4	25	16.00	[6.14; 35.69]
Al−Qattan 2011	4	35	11.43	[4.36; 26.77]
Al−Qattan 2008	0	15	0.00	[0.19; 35.03]
Ali 2007	5	94	5.32	[2.23; 12.15]
An 2013	0	27	0.00	[0.11; 22.96]
Anakwe 2010	0	21	0.00	[0.14; 27.74]
Anderson 2004	9	24	37.50	[20.80; 57.82]
Arora 2022	1	28	3.57	[0.50; 21.42]
Bach 2006	0	10	0.00	[0.28; 44.83]
Basat 2017	9	61	14.75	[7.86; 25.99]
Bauze 1999	2	10	20.00	[5.04; 54.07]
Beeres 2021	0	12	0.00	[0.24; 40.32]
Bhat 2002	11	120	9.17	[5.15; 15.80]
Botte 1992	10	137	7.30	[3.97; 13.03]
Boussakri 2014	3	28	10.71	[3.50; 28.44]
Brei−Thoma 2015	0	32	0.00	[0.09; 20.08]
Camacho 2021	0	45	0.00	[0.07; 15.14]
Campochiaro 2011	0	77	0.00	[0.04; 9.43]
Capo 2011	2	102	1.96	[0.49; 7.50]
Chafik 2011	5	24	20.83	[8.95; 41.34]
Cheah 2012	0	13	0.00	[0.22; 38.39]
Cheng 2020	2	17	11.76	[2.96; 36.83]
Choubey 2022	1	28	3.57	[0.50; 21.42]
Conolly 1973	18	260	6.92	[4.40; 10.72]
de Haseth 2015	1	9	11.11	[1.54; 49.99]
Dennison 2007	0	5	0.00	[0.50; 62.18]
Dumont 2007	0	12	0.00	[0.24; 40.32]
Duncan 1993	6	75	8.00	[3.64; 16.68]
Eglseder 1993	0	19	0.00	[0.15; 29.81]
Erken 2015	0	12	0.00	[0.24; 40.32]
Fan 2016	0	12	0.00	[0.24; 40.32]
Ficke 2018	5	47	10.64	[4.50; 23.13]
Gradl 2005	3	25	12.00	[3.92; 31.30]
Grundberg 1981	0	21	0.00	[0.14; 27.74]
Guerro 2019	2	23	8.70	[2.18; 28.88]
Henry 2008	7	108	6.48	[3.12; 12.97]
Hove 1997	1	29	3.45	[0.48; 20.79]
Huang 2018	0	24	0.00	[0.12; 25.13]
Hussain 2013	0	113	0.00	[0.03; 6.62]
Ibrahim 2022	0	17	0.00	[0.17; 32.21]
Ignacio 1999	5	25	20.00	[8.58; 39.98]
Johandi 2017	1	12	8.33	[1.16; 41.32]
Joo 2022	2	24	8.33	[2.09; 27.88]
Kilic 2015	21	67	31.34	[21.41; 43.34]
Kömürcü 2005	4	24	16.67	[6.40; 36.91]
Kucukguven 2019	4	23	17.39	[6.68; 38.23]
Kural 2020	0	11	0.00	[0.26; 42.46]
Lee 2003	1	22	4.55	[0.64; 26.15]
Li C 2016	0	66	0.00	[0.05; 10.83]
Li S 2016	0	16	0.00	[0.18; 33.56]
Liechti	0	28	0.00	[0.11; 22.32]
Lopes 1997	1	19	5.26	[0.74; 29.39]
Marshall 1978	0	7	0.00	[0.38; 53.86]
Marsland 2012	3	8	37.50	[12.54; 71.52]
Martins 2021	0	37	0.00	[0.08; 17.84]
Mattiassich 2013	4	26	15.38	[5.90; 34.54]
Matullo 2015	1	21	4.76	[0.67; 27.14]
Meena 2021	2	25	8.00	[2.01; 26.94]
Naguib 2022	1	20	5.00	[0.70; 28.22]
Nazerani 2009	0	24	0.00	[0.12; 25.13]
Nazerani 2011	4	64	6.25	[2.37; 15.50]
Page 1998	3	82	3.66	[1.18; 10.74]
Pan 2020	0	17	0.00	[0.17; 32.21]
Parmaksizoglu 2016	0	3	0.00	[0.73; 73.44]
Pelissier 2015	4	86	4.65	[1.76; 11.74]
Prunieres 2016	0	12	0.00	[0.24; 40.32]
Rabarin 2016	0	28	0.00	[0.11; 22.32]
Rajappa 2014	1	26	3.85	[0.54; 22.79]
Reissner 2012	5	32	15.62	[6.66; 32.47]
Ring 1997	0	22	0.00	[0.13; 26.81]
Rocchi 2006	1	48	2.08	[0.29; 13.36]
Saint−Cyr 2006	0	7	0.00	[0.38; 53.86]
Singh 2019	1	11	9.09	[1.26; 43.86]
Smith 1988	0	16	0.00	[0.18; 33.56]
Solari 2021	9	200	4.50	[2.36; 8.42]
Stahl 2001	13	236	5.51	[3.23; 9.25]
Starker 1995	0	68	0.00	[0.05; 10.55]
Swanson 1991	7	117	5.98	[2.88; 12.02]
Szalay 2011	0	77	0.00	[0.04; 9.43]
Tarallo 2013	1	303	0.33	[0.05; 2.30]
Tarallo 2020	1	110	0.91	[0.13; 6.17]
Tyllianakis 2010	7	20	35.00	[17.68; 57.44]
Vasudevan 2017	52	458	11.35	[8.76; 14.60]
Wang 2022	0	24	0.00	[0.12; 25.13]
Wang 2021	0	20	0.00	[0.15; 28.74]
Zhang 2010	0	65	0.00	[0.05; 10.98]
Ahmad 2022	6	190	3.16	[1.43; 6.85]
Ahmad 2006	1	38	2.63	[0.37; 16.46]
Al−Qattan 2012	2	36	5.56	[1.39; 19.67]
Al−Qattan 2005	0	73	0.00	[0.04; 9.89]
Al−Shahwanii 2021	5	26	19.23	[8.24; 38.70]
Baldwin 2021	20	556	3.60	[2.33; 5.51]
Baumgartner 2021	0	79	0.00	[0.04; 9.21]
Bennur 2021	6	80	7.50	[3.41; 15.70]
Benson 2006	7	34	20.59	[10.14; 37.32]
Cassell 1997	9	226	3.98	[2.08; 7.47]
Chul−Ho 2021	1	25	4.00	[0.56; 23.55]
Constantine 2022	781	87169	0.90	[0.84; 0.96]
Donnally 2017	6	41	14.63	[6.73; 28.96]
Egol 2010	0	44	0.00	[0.07; 15.43]
Faruqui 2012	1	50	2.00	[0.28; 12.88]
Feldman 2021	7	107	6.54	[3.15; 13.09]
Frueh 2014	1	191	0.52	[0.07; 3.62]
Fu 2006	1	98	1.02	[0.14; 6.88]
Galivanche 2021	383	16990	2.25	[2.04; 2.49]
Gereli 2010	3	30	10.00	[3.26; 26.81]
Ghosh 2013	4	30	13.33	[5.10; 30.59]
Goslings 1999	9	39	23.08	[12.47; 38.72]
Gumussuyu 2021	1	21	4.76	[0.67; 27.14]
Henry 2020	0	24	0.00	[0.12; 25.13]
Hery 2022	5	405	1.23	[0.51; 2.93]
Johnson 2021	2	89	2.25	[0.56; 8.54]
Jokhio 2021	1	200	0.50	[0.07; 3.46]
Joosten 1999	20	174	11.49	[7.54; 17.15]
Jose 2017	1	53	1.89	[0.27; 12.21]
Kadhum 2022	7	151	4.64	[2.23; 9.40]
Kim 2013	3	60	5.00	[1.62; 14.39]
Kurzen 2006	3	54	5.56	[1.80; 15.86]
Lallemand 2002	1	43	2.33	[0.33; 14.75]
Langridge 2021	3	49	6.12	[1.99; 17.33]
Lassner 2001	3	48	6.25	[2.03; 17.66]
Lattman 2011	1	245	0.41	[0.06; 2.84]
Lauder 2015	0	18	0.00	[0.16; 30.96]
Lee 2020	85	1921	4.42	[3.59; 5.44]
Levy 2021	0	90	0.00	[0.03; 8.17]
Li 2022	1	70	1.43	[0.20; 9.45]
Liu 2021	5	24	20.83	[8.95; 41.34]
Liu Q 2021	5	24	20.83	[8.95; 41.34]
Liu Y 2020	15	207	7.25	[4.42; 11.67]
Lutsky 2019	17	141	12.06	[7.63; 18.54]
Lutz 2014	16	129	12.40	[7.74; 19.29]
Mahmood 2022	456	132650	0.34	[0.31; 0.38]
McCallister 2006	2	26	7.69	[1.93; 26.07]
Meaike 2021	0	115	0.00	[0.03; 6.51]
Mehdinasab 2012	0	32	0.00	[0.09; 20.08]
Miao 2015	6	41	14.63	[6.73; 28.96]
Michael 2022	3	32	9.38	[3.06; 25.35]
Minhas 2019	31	3506	0.88	[0.62; 1.25]
Nguyen 2018	0	30	0.00	[0.10; 21.14]
Nygaard 2011	24	51	47.06	[33.90; 60.64]
Ochman 2006	7	67	10.45	[5.06; 20.33]
Ornelli 2019	0	12	0.00	[0.24; 40.32]
Platt 1995	13	124	10.48	[6.19; 17.22]
Rao 2022	0	78	0.00	[0.04; 9.32]
Raza 2021	5	50	10.00	[4.22; 21.87]
Ridley 2017	99	695	14.24	[11.84; 17.05]
Saied 2019	2	61	3.28	[0.82; 12.18]
Shakoor 2021	3	16	18.75	[6.17; 44.75]
Shetty 1997	0	102	0.00	[0.03; 7.28]
Sim 2021	3	232	1.29	[0.42; 3.93]
Skochdopole 2022	13	5894	0.22	[0.13; 0.38]
Starr 2021	2	27	7.41	[1.86; 25.25]
Stone 1998	3	140	2.14	[0.69; 6.43]
Suprock 1990	8	91	8.79	[4.46; 16.61]
Tareen 2019	14	92	15.22	[9.22; 24.07]
Terndrup 2018	21	444	4.73	[3.10; 7.14]
Teunis 2015	11	1511	0.73	[0.40; 1.31]
Thakur 2021	0	21	0.00	[0.14; 27.74]
Truntzer 2018	154	7890	1.95	[1.67; 2.28]
Van Leeuwen 2016	85	1213	7.01	[5.70; 8.59]
Wang 2021	0	11	0.00	[0.26; 42.46]
Wang 2021	0	6	0.00	[0.43; 57.72]
Wei 2021	57	39275	0.15	[0.11; 0.19]
Weinand 2014	67	401	16.71	[13.37; 20.68]
Yim 2004	2	26	7.69	[1.93; 26.07]
Zhou 2008	3	72	4.17	[1.35; 12.14]
Aydin 2010	24	818	2.93	[1.97; 4.34]
Azzopardi 2005	1	30	3.33	[0.47; 20.20]
Berwald 2014	1	73	1.37	[0.19; 9.09]
Chung 2019	30	183	16.39	[11.71; 22.48]
Costa 2022	4	245	1.63	[0.61; 4.27]
Costa 2014	32	461	6.94	[4.95; 9.65]
Dias 2020	2	203	0.99	[0.25; 3.85]
Egol 2006	21	118	17.80	[11.90; 25.76]
Foldhazy 2010	4	22	18.18	[6.99; 39.64]
Grossman 1981	3	265	1.13	[0.37; 3.45]
Hargreaves 2004	12	56	21.43	[12.59; 34.05]
Horton 2003	6	15	40.00	[19.18; 65.19]
Hove 2010	19	70	27.14	[18.03; 38.68]
Khan 2022	21	122	17.21	[11.50; 24.96]
Kivi 2011	15	99	15.15	[9.35; 23.62]
Lawson 2021	0	80	0.00	[0.04; 9.10]
Lee 2022	2	25	8.00	[2.01; 26.94]
Lundqvist 2022	3	147	2.04	[0.66; 6.13]
Maradei−Pereira 2021	14	220	6.36	[3.80; 10.46]
Mishra 2021	3	62	4.84	[1.57; 13.96]
Noor 2021	7	56	12.50	[6.08; 23.98]
Peacock 1988	1	87	1.15	[0.16; 7.71]
Rafique 2006	12	60	20.00	[11.72; 32.00]
Supichyangur 2022	1	23	4.35	[0.61; 25.22]
Werber 2003	6	50	12.00	[5.49; 24.24]
Whittaker 2005	17	157	10.83	[6.84; 16.73]
Zettl 2009	1	120	0.83	[0.12; 5.67]
Davies 2020	41	983	4.17	[3.09; 5.62]
Bednar 2004	2	12	16.67	[4.20; 47.72]
Dwyer 2017	6	25	24.00	[11.20; 44.16]
**Common effect model**			**2.07**	**[2.00; 2.15]**
**Random effects model**			**5.01**	**[4.01; 6.24]**

Heterogeneity: $I^2 = 97\%$, $t^2 = 2.1613$, $c^2_{200} = 5927.39$ ($p = 0$)

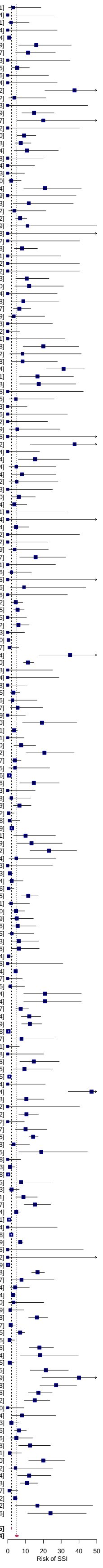

Supplementary Figure 3. Meta-analysis – methodological subgroup analyses

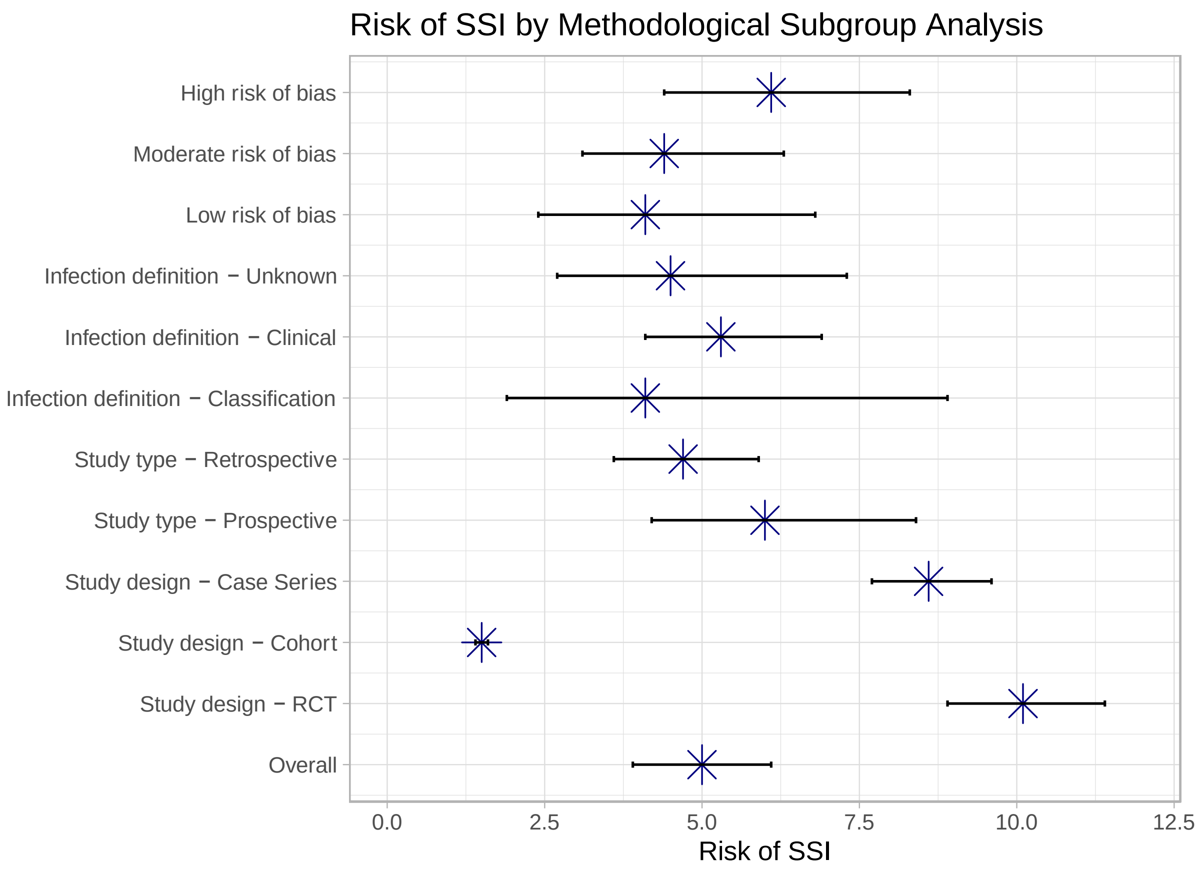

Supplementary Figure 4. Meta-analysis – subgroup analysis by study design

Study	Risk of SSI	95% C.I.
**Studydesign = Case series**		
Abouelela 2020	3.03	[0.43; 18.61]
Abulsoud 2021	0.00	[0.13; 25.94]
Adkison 1982	1.85	[0.26; 12.01]
Afshar 2020	0.00	[0.14; 27.74]
Aigner 2014	0.83	[0.37; 1.84]
Akmaz 2003	16.00	[6.14; 35.69]
Al−Qattan 2011	11.43	[4.36; 26.77]
Al−Qattan 2008	0.00	[0.19; 35.03]
Ali 2007	5.32	[2.23; 12.15]
An 2013	0.00	[0.11; 22.96]
Anakwe 2010	0.00	[0.14; 27.74]
Anderson 2004	37.50	[20.80; 57.82]
Arora 2022	3.57	[0.50; 21.42]
Bach 2006	0.00	[0.28; 44.83]
Basat 2017	14.75	[7.86; 25.99]
Bauze 1999	20.00	[5.04; 54.07]
Beeres 2021	0.00	[0.24; 40.32]
Bhat 2002	9.17	[5.15; 15.80]
Botte 1992	7.30	[3.97; 13.03]
Boussakri 2014	10.71	[3.50; 28.44]
Brei−Thoma 2015	0.00	[0.09; 20.08]
Camacho 2021	0.00	[0.07; 15.14]
Campochiaro 2011	0.00	[0.04; 9.43]
Capo 2011	1.96	[0.49; 7.50]
Chafik 2011	20.83	[8.95; 41.34]
Cheah 2012	0.00	[0.22; 38.39]
Cheng 2020	11.76	[2.96; 36.83]
Choubey 2022	3.57	[0.50; 21.42]
Conolly 1973	6.92	[4.40; 10.72]
de Haseth 2015	11.11	[1.54; 49.99]
Dennison 2007	0.00	[0.50; 62.18]
Dumont 2007	0.00	[0.24; 40.32]
Duncan 1993	8.00	[3.64; 16.68]
Eglseder 1993	0.00	[0.15; 29.81]
Erken 2015	0.00	[0.24; 40.32]
Fan 2016	0.00	[0.24; 40.32]
Ficke 2018	10.64	[4.50; 23.13]
Gradl 2005	12.00	[3.92; 31.30]
Grundberg 1981	0.00	[0.14; 27.74]
Guerro 2019	8.70	[2.18; 28.88]
Henry 2008	6.48	[3.12; 12.97]
Hove 1997	3.45	[0.48; 20.79]
Huang 2018	0.00	[0.12; 25.13]
Hussain 2013	0.00	[0.03; 6.62]
Ibrahim 2022	0.00	[0.17; 32.21]
Ignacio 1999	20.00	[8.58; 39.98]
Johandi 2017	8.33	[1.16; 41.32]
Joo 2022	8.33	[2.09; 27.88]
Kilic 2015	31.34	[21.41; 43.34]
Kömürcü 2005	16.67	[6.40; 36.91]
Kucukguven 2019	17.39	[6.68; 38.23]
Kural 2020	0.00	[0.26; 42.46]
Lee 2003	4.55	[0.64; 26.15]
Li C 2016	0.00	[0.05; 10.83]
Li S 2016	0.00	[0.18; 33.56]
Liechti	0.00	[0.11; 22.32]
Lopes 1997	5.26	[0.74; 29.39]
Marshall 1978	0.00	[0.38; 53.86]
Marsland 2012	37.50	[12.54; 71.52]
Martins 2021	0.00	[0.08; 17.84]
Mattiassich 2013	15.38	[5.90; 34.54]
Matullo 2015	4.76	[0.67; 27.14]
Meena 2021	8.00	[2.01; 26.94]
Naguib 2022	5.00	[0.70; 28.22]
Nazerani 2009	0.00	[0.12; 25.13]
Nazerani 2011	6.25	[2.37; 15.50]
Page 1998	3.66	[1.18; 10.74]
Pan 2020	0.00	[0.17; 32.21]
Parmaksizoglu 2016	0.00	[0.73; 73.44]
Pelissier 2015	4.65	[1.76; 11.74]
Prunieres 2016	0.00	[0.24; 40.32]
Rabarin 2016	0.00	[0.11; 22.32]
Rajappa 2014	3.85	[0.54; 22.79]
Reissner 2012	15.62	[6.66; 32.47]
Ring 1997	0.00	[0.13; 26.81]
Rocchi 2006	2.08	[0.29; 13.36]
Saint−Cyr 2006	0.00	[0.38; 53.86]
Singh 2019	9.09	[1.26; 43.86]
Smith 1988	0.00	[0.18; 33.56]
Solari 2021	4.50	[2.36; 8.42]
Stahl 2001	5.51	[3.23; 9.25]
Starker 1995	0.00	[0.05; 10.55]
Swanson 1991	5.98	[2.88; 12.02]
Szalay 2011	0.00	[0.04; 9.43]
Tarallo 2013	0.33	[0.05; 2.30]
Tarallo 2020	0.91	[0.13; 6.17]
Tyllianakis 2010	35.00	[17.68; 57.44]
Vasudevan 2017	11.35	[8.76; 14.60]
Wang 2022	0.00	[0.12; 25.13]
Wang 2021	0.00	[0.15; 28.74]
Zhang 2010	0.00	[0.05; 10.98]
**Common effect model**	**8.60**	**[7.69; 9.61]**
**Random effects model**	**8.60**	**[7.69; 9.61]**
Heterogeneity: $I^2 = 61\%$, $\tau^2 = 0$, $\chi^2_{90} = 232.39$ ($p < 0.01$)		
**Studydesign = Cohort**		
Ahmad 2022	3.16	[1.43; 6.85]
Ahmad 2006	2.63	[0.37; 16.46]
Al−Qattan 2012	5.56	[1.39; 19.67]
Al−Qattan 2005	0.00	[0.04; 9.89]
Al−Shahwanii 2021	19.23	[8.24; 38.70]
Baldwin 2021	3.60	[2.33; 5.51]
Baumgartner 2021	0.00	[0.04; 9.21]
Bennur 2021	7.50	[3.41; 15.70]
Benson 2006	20.59	[10.14; 37.32]
Cassell 1997	3.98	[2.08; 7.47]
Chul−Ho 2021	4.00	[0.56; 23.55]
Constantine 2022	0.90	[0.84; 0.96]
Donnally 2017	14.63	[6.73; 28.96]
Egol 2010	0.00	[0.07; 15.43]
Faruqui 2012	2.00	[0.28; 12.88]
Feldman 2021	6.54	[3.15; 13.09]
Frueh 2014	0.52	[0.07; 3.62]
Fu 2006	1.02	[0.14; 6.88]
Galivanche 2021	2.25	[2.04; 2.49]
Gereli 2010	10.00	[3.26; 26.81]
Ghosh 2013	13.33	[5.10; 30.59]
Goslings 1999	23.08	[12.47; 38.72]
Gumussuyu 2021	4.76	[0.67; 27.14]
Henry 2020	0.00	[0.12; 25.13]
Hery 2022	1.23	[0.51; 2.93]
Johnson 2021	2.25	[0.56; 8.54]
Jokhio 2021	0.50	[0.07; 3.46]
Joosten 1999	11.49	[7.54; 17.15]
Jose 2017	1.89	[0.27; 12.21]
Kadhum 2022	4.64	[2.23; 9.40]
Kim 2013	5.00	[1.62; 14.39]
Kurzen 2006	5.56	[1.80; 15.86]
Lallemand 2002	2.33	[0.33; 14.75]
Langridge 2021	6.12	[1.99; 17.33]
Lassner 2001	6.25	[2.03; 17.66]
Lattman 2011	0.41	[0.06; 2.84]
Lauder 2015	0.00	[0.16; 30.96]
Lee 2020	4.42	[3.59; 5.44]
Levy 2021	0.00	[0.03; 8.17]
Li 2022	1.43	[0.20; 9.45]
Liu 2021	20.83	[8.95; 41.34]
Liu Q 2021	20.83	[8.95; 41.34]
Liu Y 2020	7.25	[4.42; 11.67]
Lutsky 2019	12.06	[7.63; 18.54]
Lutz 2014	12.40	[7.74; 19.29]
Mahmood 2022	0.34	[0.31; 0.38]
McCallister 2006	7.69	[1.93; 26.07]
Meaike 2021	0.00	[0.03; 6.51]
Mehdinasab 2012	0.00	[0.09; 20.08]
Miao 2015	14.63	[6.73; 28.96]
Michael 2022	9.38	[3.06; 25.35]
Minhas 2019	0.88	[0.62; 1.25]
Nguyen 2018	0.00	[0.10; 21.14]
Nygaard 2011	47.06	[33.90; 60.64]
Ochman 2006	10.45	[5.06; 20.33]
Ornelli 2019	0.00	[0.24; 40.32]
Platt 1995	10.48	[6.19; 17.22]
Rao 2022	0.00	[0.04; 9.32]
Raza 2021	10.00	[4.22; 21.87]
Ridley 2017	14.24	[11.84; 17.05]
Saied 2019	3.28	[0.82; 12.18]
Shakoor 2021	18.75	[6.17; 44.75]
Shetty 1997	0.00	[0.03; 7.28]
Sim 2021	1.29	[0.42; 3.93]
Skochdopole 2022	0.22	[0.13; 0.38]
Starr 2021	7.41	[1.86; 25.25]
Stone 1998	2.14	[0.69; 6.43]
Suprock 1990	8.79	[4.46; 16.61]
Tareen 2019	15.22	[9.22; 24.07]
Terndrup 2018	4.73	[3.10; 7.14]
Teunis 2015	0.73	[0.40; 1.31]
Thakur 2021	0.00	[0.14; 27.74]
Truntzer 2018	1.95	[1.67; 2.28]
Van Leeuwen 2016	7.01	[5.70; 8.59]
Wang 2021	0.00	[0.26; 42.46]
Wang 2021	0.00	[0.43; 57.72]
Wei 2021	0.15	[0.11; 0.19]
Weinand 2014	16.71	[13.37; 20.68]
Yim 2004	7.69	[1.93; 26.07]
Zhou 2008	4.17	[1.35; 12.14]
Davies 2020	4.17	[3.09; 5.62]
**Common effect model**	**1.52**	**[1.46; 1.58]**
**Random effects model**	**1.52**	**[1.46; 1.58]**
Heterogeneity: $I^2 = 98\%$, $\tau^2 = 0$, $\chi^2_{80} = 3998.12$ ($p = 0$)		
**Studydesign = RCT**		
Aydin 2010	2.93	[1.97; 4.34]
Azzopardi 2005	3.33	[0.47; 20.20]
Berwald 2014	1.37	[0.19; 9.09]
Chung 2019	16.39	[11.71; 22.48]
Costa 2022	1.63	[0.61; 4.27]
Costa 2014	6.94	[4.95; 9.65]
Dias 2020	0.99	[0.25; 3.85]
Egol 2006	17.80	[11.90; 25.76]
Foldhazy 2010	18.18	[6.99; 39.64]
Grossman 1981	1.13	[0.37; 3.45]
Hargreaves 2004	21.43	[12.59; 34.05]
Horton 2003	40.00	[19.18; 65.19]
Hove 2010	27.14	[18.03; 38.68]
Khan 2022	17.21	[11.50; 24.96]
Kivi 2011	15.15	[9.35; 23.62]
Lawson 2021	0.00	[0.04; 9.10]
Lee 2022	8.00	[2.01; 26.94]
Lundqvist 2022	2.04	[0.66; 6.13]
Maradei−Pereira 2021	6.36	[3.80; 10.46]
Mishra 2021	4.84	[1.57; 13.96]
Noor 2021	12.50	[6.08; 23.98]
Peacock 1988	1.15	[0.16; 7.71]
Rafique 2006	20.00	[11.72; 32.00]
Supichyangur 2022	4.35	[0.61; 25.22]
Werber 2003	12.00	[5.49; 24.24]
Whittaker 2005	10.83	[6.84; 16.73]
Zettl 2009	0.83	[0.12; 5.67]
**Common effect model**	**10.11**	**[8.99; 11.36]**
**Random effects model**	**10.11**	**[8.99; 11.36]**
Heterogeneity: $I^2 = 87\%$, $\tau^2 = 0$, $\chi^2_{26} = 194.13$ ($p < 0.01$)		

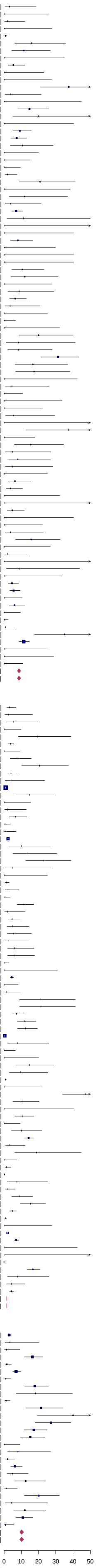

Test for subgroup differences (common effect): $\chi^2_2 = 1460.93$, df = 2 ($p < 0.01$)
Test for subgroup differences (random effects): $\chi^2_2 = 1460.93$, df = 2 ($p < 0.01$)

Supplementary Figure 5. Meta-analysis – subgroup analysis by study type

Study	Risk of SSI	95% C.I.
**Studytype = Retrospective**		
Abouelela 2020	3.03	[0.43; 18.61]
Adkison 1982	1.85	[0.26; 12.01]
Aigner 2014	0.83	[0.37; 1.84]
Akmaz 2003	16.00	[6.14; 35.69]
Al−Qattan 2011	11.43	[4.36; 26.77]
An 2013	0.00	[0.11; 22.96]
Anderson 2004	37.50	[20.80; 57.82]
Arora 2022	3.57	[0.50; 21.42]
Bach 2006	0.00	[0.28; 44.83]
Basat 2017	14.75	[7.86; 25.99]
Bauze 1999	20.00	[5.04; 54.07]
Beeres 2021	0.00	[0.24; 40.32]
Bhat 2002	9.17	[5.15; 15.80]
Botte 1992	7.30	[3.97; 13.03]
Boussakri 2014	10.71	[3.50; 28.44]
Brei−Thoma 2015	0.00	[0.09; 20.08]
Camacho 2021	0.00	[0.07; 15.14]
Campochiaro 2011	0.00	[0.04; 9.43]
Capo 2011	1.96	[0.49; 7.50]
Cheah 2012	0.00	[0.22; 38.39]
Cheng 2020	11.76	[2.96; 36.83]
Conolly 1973	6.92	[4.40; 10.72]
de Haseth 2015	11.11	[1.54; 49.99]
Dennison 2007	0.00	[0.50; 62.18]
Dumont 2007	0.00	[0.24; 40.32]
Duncan 1993	8.00	[3.64; 16.68]
Eglseder 1993	0.00	[0.15; 29.81]
Erken 2015	0.00	[0.24; 40.32]
Fan 2016	0.00	[0.24; 40.32]
Ficke 2018	10.64	[4.50; 23.13]
Grundberg 1981	0.00	[0.14; 27.74]
Guerro 2019	8.70	[2.18; 28.88]
Henry 2008	6.48	[3.12; 12.97]
Hove 1997	3.45	[0.48; 20.79]
Huang 2018	0.00	[0.12; 25.13]
Hussain 2013	0.00	[0.03; 6.62]
Ibrahim 2022	0.00	[0.17; 32.21]
Johandi 2017	8.33	[1.16; 41.32]
Joo 2022	8.33	[2.09; 27.88]
Kilic 2015	31.34	[21.41; 43.34]
Kömürcü 2005	16.67	[6.40; 36.91]
Kural 2020	0.00	[0.26; 42.46]
Lee 2003	4.55	[0.64; 26.15]
Li S 2016	0.00	[0.18; 33.56]
Lopes 1997	5.26	[0.74; 29.39]
Marshall 1978	0.00	[0.38; 53.86]
Marsland 2012	37.50	[12.54; 71.52]
Martins 2021	0.00	[0.08; 17.84]
Mattiassich 2013	15.38	[5.90; 34.54]
Matullo 2015	4.76	[0.67; 27.14]
Meena 2021	8.00	[2.01; 26.94]
Nazerani 2009	0.00	[0.12; 25.13]
Nazerani 2011	6.25	[2.37; 15.50]
Page 1998	3.66	[1.18; 10.74]
Parmaksizoglu 2016	0.00	[0.73; 73.44]
Pelissier 2015	4.65	[1.76; 11.74]
Rabarin 2016	0.00	[0.11; 22.32]
Reissner 2012	15.62	[6.66; 32.47]
Rocchi 2006	2.08	[0.29; 13.36]
Saint−Cyr 2006	0.00	[0.38; 53.86]
Singh 2019	9.09	[1.26; 43.86]
Smith 1988	0.00	[0.18; 33.56]
Solari 2021	4.50	[2.36; 8.42]
Stahl 2001	5.51	[3.23; 9.25]
Swanson 1991	5.98	[2.88; 12.02]
Szalay 2011	0.00	[0.04; 9.43]
Tarallo 2013	0.33	[0.05; 2.30]
Tarallo 2020	0.91	[0.13; 6.17]
Tyllianakis 2010	35.00	[17.68; 57.44]
Vasudevan 2017	11.35	[8.76; 14.60]
Wang 2022	0.00	[0.12; 25.13]
Wang 2021	0.00	[0.15; 28.74]
Ahmad 2022	3.16	[1.43; 6.85]
Ahmad 2006	2.63	[0.37; 16.46]
Al−Qattan 2012	5.56	[1.39; 19.67]
Al−Qattan 2005	0.00	[0.04; 9.89]
Baldwin 2021	3.60	[2.33; 5.51]
Baumgartner 2021	0.00	[0.04; 9.21]
Benson 2006	20.59	[10.14; 37.32]
Chul−Ho 2021	4.00	[0.56; 23.55]
Constantine 2022	0.90	[0.84; 0.96]
Donnally 2017	14.63	[6.73; 28.96]
Egol 2010	0.00	[0.07; 15.43]
Faruqui 2012	2.00	[0.28; 12.88]
Feldman 2021	6.54	[3.15; 13.09]
Frueh 2014	0.52	[0.07; 3.62]
Galivanche 2021	2.25	[2.04; 2.49]
Gereli 2010	10.00	[3.26; 26.81]
Gumussuyu 2021	4.76	[0.67; 27.14]
Henry 2020	0.00	[0.12; 25.13]
Johnson 2021	2.25	[0.56; 8.54]
Kadhum 2022	4.64	[2.23; 9.40]
Kim 2013	5.00	[1.62; 14.39]
Kurzen 2006	5.56	[1.80; 15.86]
Lallemand 2002	2.33	[0.33; 14.75]
Langridge 2021	6.12	[1.99; 17.33]
Lassner 2001	6.25	[2.03; 17.66]
Lee 2020	4.42	[3.59; 5.44]
Levy 2021	0.00	[0.03; 8.17]
Li 2022	1.43	[0.20; 9.45]
Liu 2021	20.83	[8.95; 41.34]
Liu Q 2021	20.83	[8.95; 41.34]
Liu Y 2020	7.25	[4.42; 11.67]
Mahmood 2022	0.34	[0.31; 0.38]
Meaike 2021	0.00	[0.03; 6.51]
Minhas 2019	0.88	[0.62; 1.25]
Nguyen 2018	0.00	[0.10; 21.14]
Nygaard 2011	47.06	[33.90; 60.64]
Ochman 2006	10.45	[5.06; 20.33]
Ornelli 2019	0.00	[0.24; 40.32]
Rao 2022	0.00	[0.04; 9.32]
Raza 2021	10.00	[4.22; 21.87]
Ridley 2017	14.24	[11.84; 17.05]
Shetty 1997	0.00	[0.03; 7.28]
Sim 2021	1.29	[0.42; 3.93]
Skochdopole 2022	0.22	[0.13; 0.38]
Starr 2021	7.41	[1.86; 25.25]
Stone 1998	2.14	[0.69; 6.43]
Tareen 2019	15.22	[9.22; 24.07]
Terndrup 2018	4.73	[3.10; 7.14]
Teunis 2015	0.73	[0.40; 1.31]
Truntzer 2018	1.95	[1.67; 2.28]
Van Leeuwen 2016	7.01	[5.70; 8.59]
Wang 2021	0.00	[0.26; 42.46]
Wang 2021	0.00	[0.43; 57.72]
Wei 2021	0.15	[0.11; 0.19]
Weinand 2014	16.71	[13.37; 20.68]
Yim 2004	7.69	[1.93; 26.07]
Zhou 2008	4.17	[1.35; 12.14]
Bednar 2004	16.67	[4.20; 47.72]
Dwyer 2017	24.00	[11.20; 44.16]
**Common effect model**	**1.63**	**[1.57; 1.69]**
**Random effects model**	**4.65**	**[3.59; 6.01]**
Heterogeneity: $I^2 = 97\%$, $t^2 = 1.8124$, $c^2_{130} = 4476.02$ (p = 0)		
**Studytype = Prospective**		
Abulsoud 2021	0.00	[0.13; 25.94]
Afshar 2020	0.00	[0.14; 27.74]
Al−Qattan 2008	0.00	[0.19; 35.03]
Ali 2007	5.32	[2.23; 12.15]
Anakwe 2010	0.00	[0.14; 27.74]
Chafik 2011	20.83	[8.95; 41.34]
Choubey 2022	3.57	[0.50; 21.42]
Gradl 2005	12.00	[3.92; 31.30]
Ignacio 1999	20.00	[8.58; 39.98]
Kucukguven 2019	17.39	[6.68; 38.23]
Li C 2016	0.00	[0.05; 10.83]
Liechti	0.00	[0.11; 22.32]
Naguib 2022	5.00	[0.70; 28.22]
Pan 2020	0.00	[0.17; 32.21]
Prunieres 2016	0.00	[0.24; 40.32]
Rajappa 2014	3.85	[0.54; 22.79]
Ring 1997	0.00	[0.13; 26.81]
Starker 1995	0.00	[0.05; 10.55]
Zhang 2010	0.00	[0.05; 10.98]
Al−Shahwanii 2021	19.23	[8.24; 38.70]
Bennur 2021	7.50	[3.41; 15.70]
Cassell 1997	3.98	[2.08; 7.47]
Fu 2006	1.02	[0.14; 6.88]
Ghosh 2013	13.33	[5.10; 30.59]
Goslings 1999	23.08	[12.47; 38.72]
Hery 2022	1.23	[0.51; 2.93]
Jokhio 2021	0.50	[0.07; 3.46]
Joosten 1999	11.49	[7.54; 17.15]
Jose 2017	1.89	[0.27; 12.21]
Lattman 2011	0.41	[0.06; 2.84]
Lauder 2015	0.00	[0.16; 30.96]
Lutsky 2019	12.06	[7.63; 18.54]
Lutz 2014	12.40	[7.74; 19.29]
McCallister 2006	7.69	[1.93; 26.07]
Mehdinasab 2012	0.00	[0.09; 20.08]
Miao 2015	14.63	[6.73; 28.96]
Michael 2022	9.38	[3.06; 25.35]
Platt 1995	10.48	[6.19; 17.22]
Saied 2019	3.28	[0.82; 12.18]
Shakoor 2021	18.75	[6.17; 44.75]
Suprock 1990	8.79	[4.46; 16.61]
Thakur 2021	0.00	[0.14; 27.74]
Aydin 2010	2.93	[1.97; 4.34]
Azzopardi 2005	3.33	[0.47; 20.20]
Berwald 2014	1.37	[0.19; 9.09]
Chung 2019	16.39	[11.71; 22.48]
Costa 2022	1.63	[0.61; 4.27]
Costa 2014	6.94	[4.95; 9.65]
Dias 2020	0.99	[0.25; 3.85]
Egol 2006	17.80	[11.90; 25.76]
Foldhazy 2010	18.18	[6.99; 39.64]
Grossman 1981	1.13	[0.37; 3.45]
Hargreaves 2004	21.43	[12.59; 34.05]
Horton 2003	40.00	[19.18; 65.19]
Hove 2010	27.14	[18.03; 38.68]
Khan 2022	17.21	[11.50; 24.96]
Kivi 2011	15.15	[9.35; 23.62]
Lawson 2021	0.00	[0.04; 9.10]
Lee 2022	8.00	[2.01; 26.94]
Lundqvist 2022	2.04	[0.66; 6.13]
Maradei−Pereira 2021	6.36	[3.80; 10.46]
Mishra 2021	4.84	[1.57; 13.96]
Noor 2021	12.50	[6.08; 23.98]
Peacock 1988	1.15	[0.16; 7.71]
Rafique 2006	20.00	[11.72; 32.00]
Supichyangur 2022	4.35	[0.61; 25.22]
Werber 2003	12.00	[5.49; 24.24]
Whittaker 2005	10.83	[6.84; 16.73]
Zettl 2009	0.83	[0.12; 5.67]
Davies 2020	4.17	[3.09; 5.62]
**Common effect model**	**8.89**	**[8.14; 9.71]**
**Random effects model**	**5.96**	**[4.23; 8.34]**
Heterogeneity: $I^2 = 79\%$, $t^2 = 1.8124$, $c^2_{69} = 335.73$ (p < 0.01)		

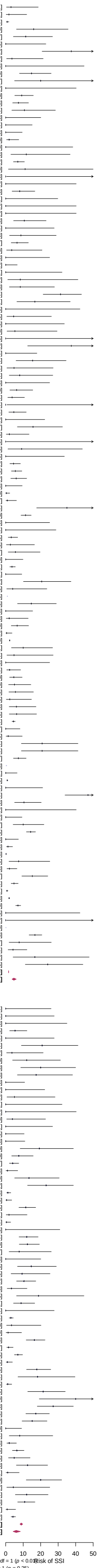

Test for subgroup differences (common effect): $c^2_1 = 1115.64$, df = 1 (p < 0.01)
Test for subgroup differences (random effects): $c^2_1 = 1.30$, df = 1 (p = 0.25)

Supplementary Figure 6. Meta-analysis – subgroup analysis by infection definition

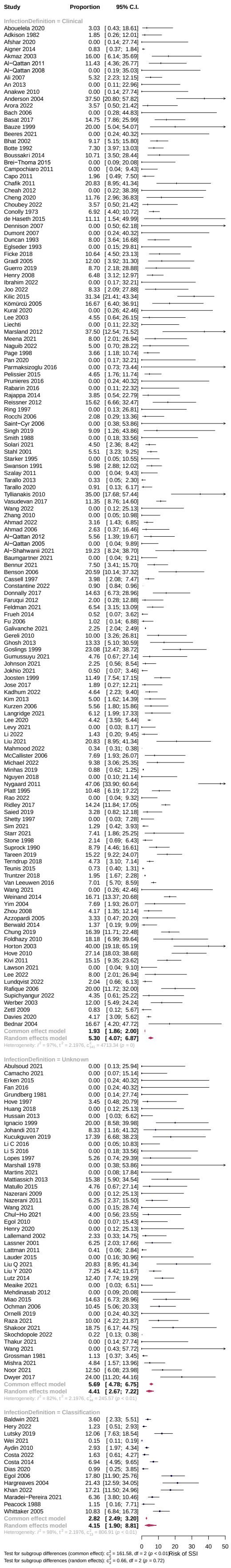

Supplementary Figure 7. Meta-analysis – subgroup analysis by risk of bias

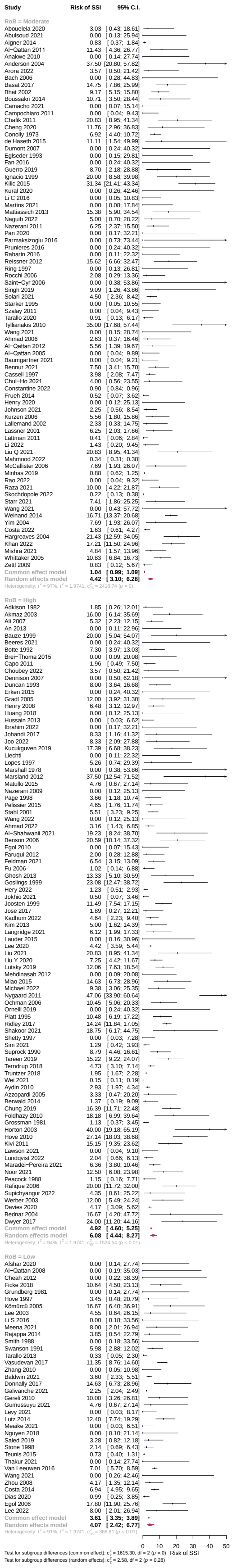

Supplementary Figure 8. Meta-analysis – clinical subgroup analyses

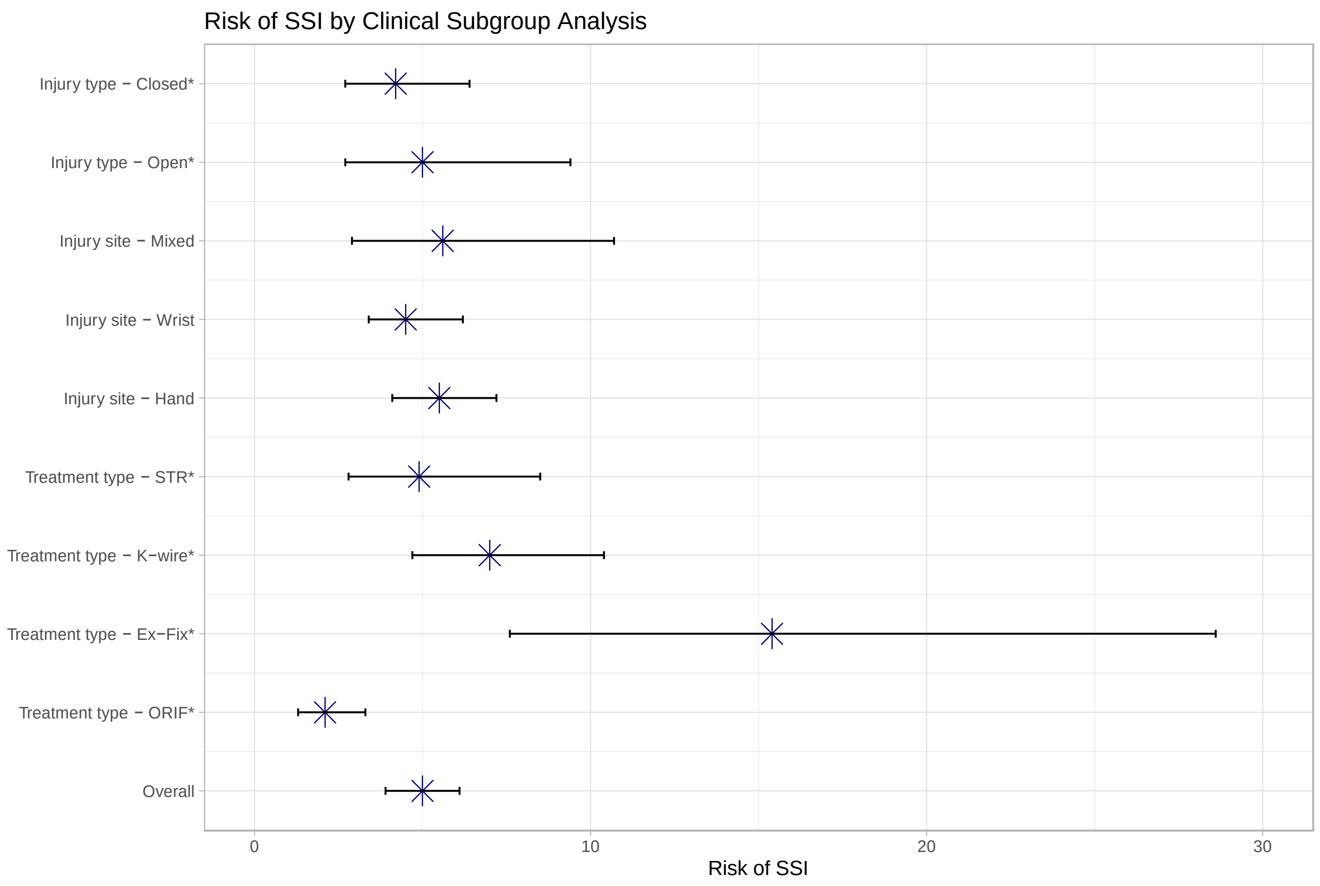

Supplementary Figure 9. Meta-analysis – subgroup analysis by injury site

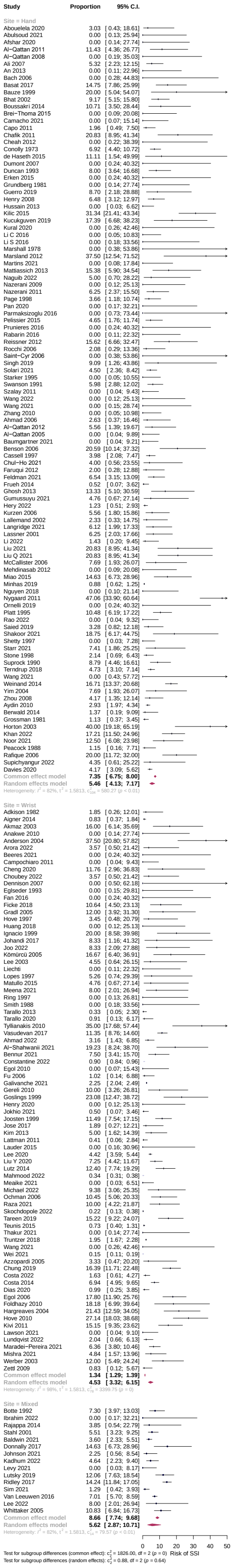

Supplementary Figure 10. Meta-analysis – subgroup analysis by injury type

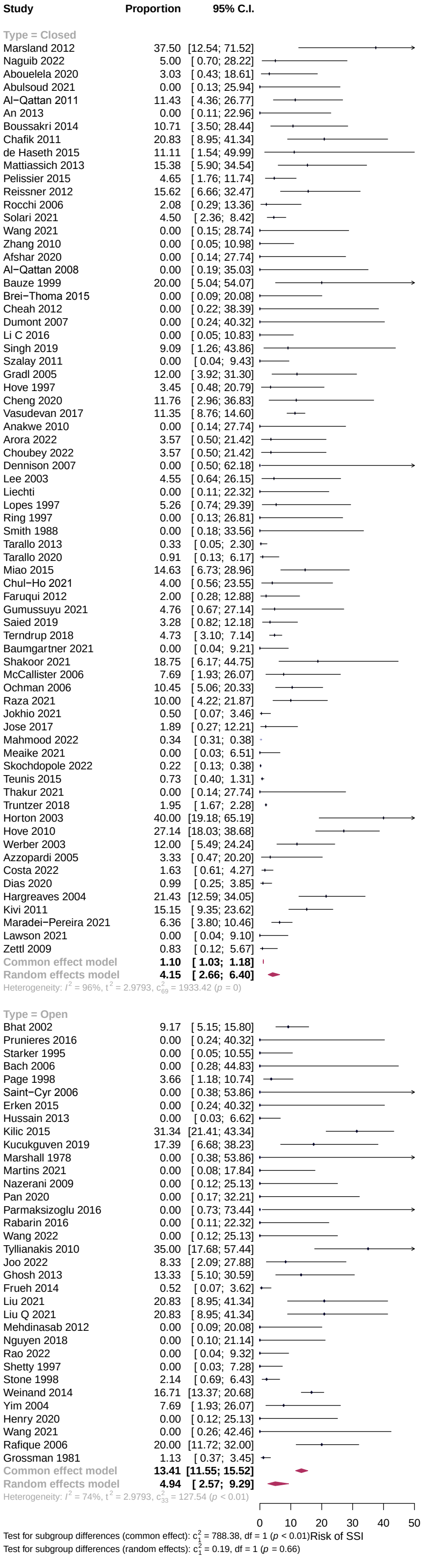

Supplementary Figure 11. Meta-analysis – subgroup analysis by intervention

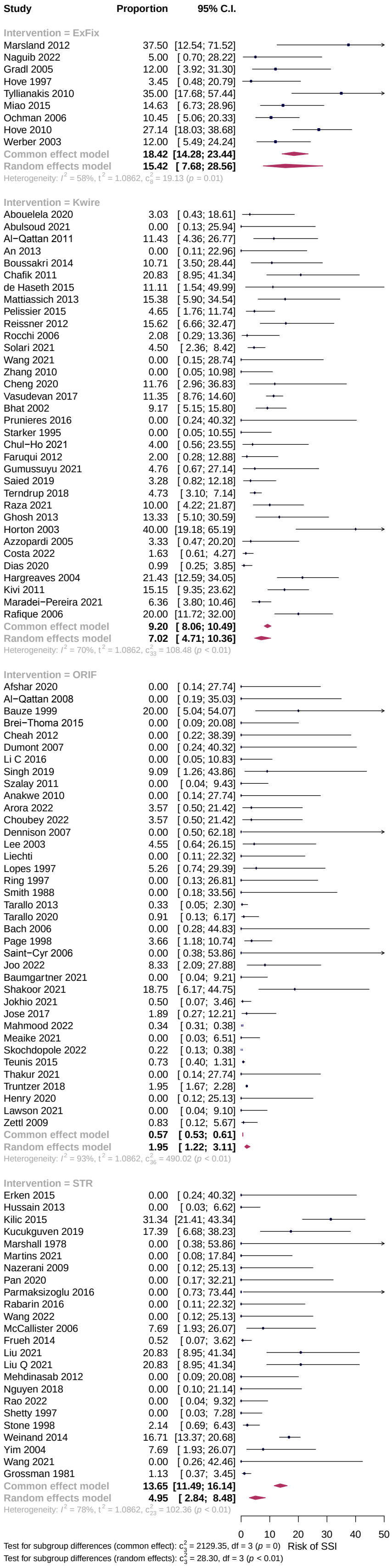

Supplementary Figure 12. Meta-analysis – funnel plot

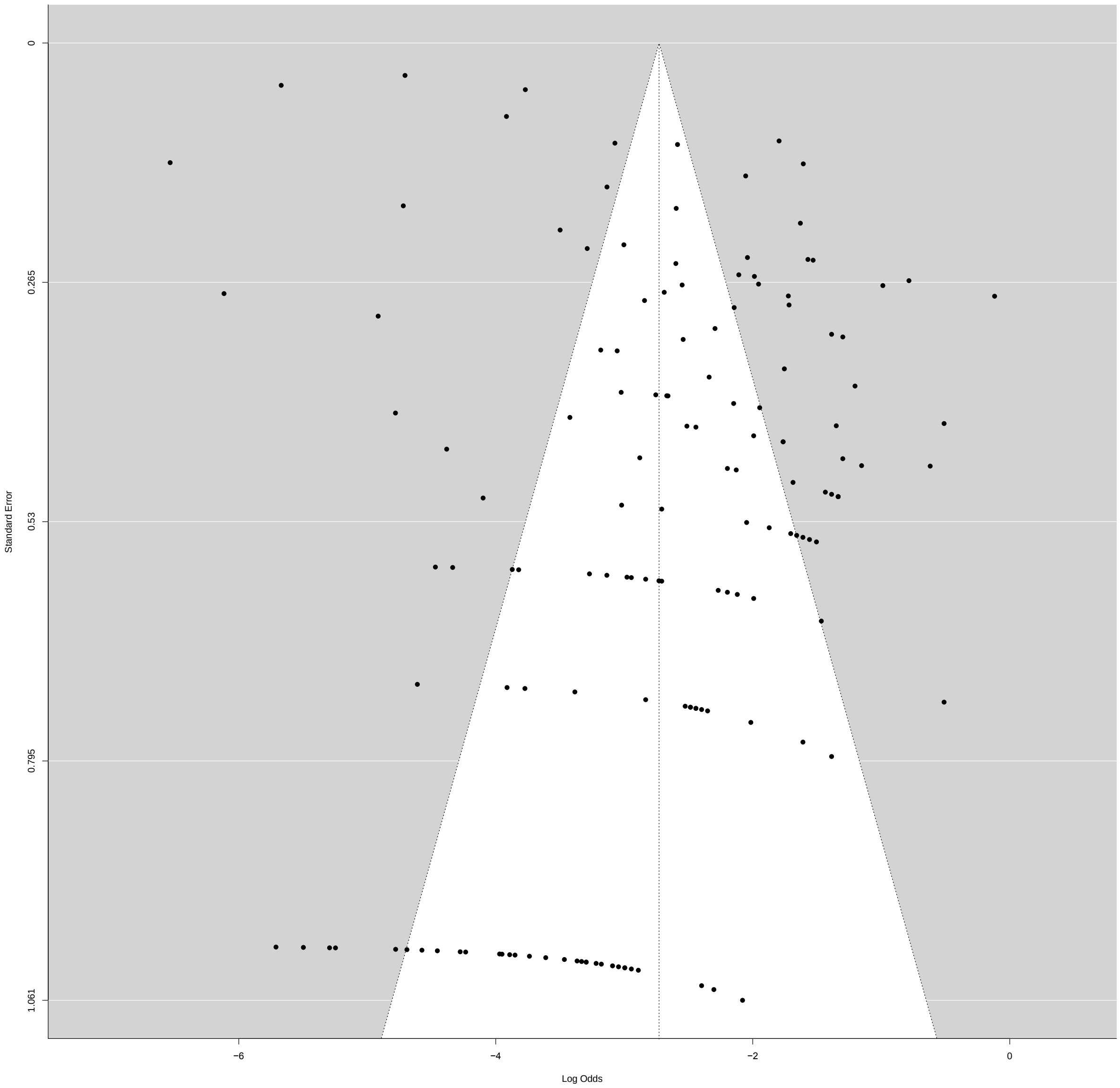
Standard Error
0
0.265
0.53
0.795
1.061
−6
−4
−2
0
Log Odds

Supplementary File 1 - Search Strategies

Search Name: Hand trauma SSI
Date Run: 16/08/2021 14:21:06
Comment:

ID	Search	Hits
#1	hand	36217
#2	trauma	20149
#3	injury	53872
#4	infection	98211
#5	#2 OR #3	66688
#6	#1 and #5	3624
#7	#4 and #6	857

Strategy 1063692/285

See full search strategy

#	Database	Search term	Results
285	CINAHL	(infect*).ti,ab AND (((((hand*).ti,ab OR (finger*).ti,ab OR (thumb*).ti,ab OR (wrist*).ti,ab OR (phalang*).ti,ab OR (metacarpal*).ti,ab OR (carpal*).ti,ab OR (radius*).ti,ab OR (ulna*).ti,ab) AND ((nerve*).ti,ab OR (neuro*).ti,ab OR (vasc*).ti,ab OR (arter*).ti,ab OR (vessel*).ti,ab OR (tendon*).ti,ab OR (ligament*).ti,ab OR (joint*).ti,ab OR (capsul*).ti,ab OR (volar*).ti,ab OR (musc*).ti,ab)) AND ((trauma*).ti,ab OR (injur*).ti,ab OR (lacerat*).ti,ab OR (fracture*).ti,ab OR (crush*).ti,ab OR (wound*).ti,ab)) AND ((surg*).ti,ab OR (operati*).ti,ab OR (intervent*).ti,ab OR (procedur*).ti,ab OR (repair*).ti,ab OR (fix*).ti,ab OR (reconstruct*).ti,ab OR (debride*).ti,ab OR (closur*).ti,ab OR (dressing*).ti,ab))	490

Contents 50 of 490 results on CINAHL - (infect*).ti,ab AND (((((hand*).ti,ab OR (finger*).ti,ab OR (thumb*).ti,ab OR (wrist*).ti,ab OR (phalang*).ti,ab OR (metacarpal*).ti,ab OR (carpal*).ti,ab OR (radius*).ti,ab OR (ulna*).ti,ab) AND ((nerve*).ti,ab OR (neuro*).ti,ab OR (vasc*).ti,ab OR (arter*).ti,ab OR (vessel*).ti,ab OR (tendon*).ti,ab OR (ligament*).ti,ab OR (joint*).ti,ab OR (capsul*).ti,ab OR (volar*).ti,ab OR (musc*).ti,ab)) AND ((trauma*).ti,ab OR (injur*).ti,ab OR (lacerat*).ti,ab OR (fracture*).ti,ab OR (crush*).ti,ab OR (wound*).ti,ab)) AND ((surg*).ti,ab OR (operati*).ti,ab OR (intervent*).ti,ab OR (procedur*).ti,ab OR (repair*).ti,ab OR (fix*).ti,ab OR (reconstruct*).ti,ab OR (debride*).ti,ab OR (closur*).ti,ab OR (dressing*).ti,ab))

1. Allograft Interposition Bone Graft for First Metatarsal Phalangeal Arthrodesis: Salvage After Bone Loss and Shortening of the First Ray. .... Page 4

2. Clinical efficacy of closed reduction and percutaneous parallel K-wire interlocking fixation of first metacarpal base fracture. .... Page 4

3. A Practical Approach to the Management of Digital Ulcers in Patients With Systemic Sclerosis: A Narrative Review. .... Page 5

4. Inclusion of Olecranon Osteotomy With the Posterior Approach for Fixation of Distal Humerus Fractures (OTA/AO 13) Does Not Increase Surgical Complications. .... Page 6

5. Antegrade intramedullary fixation for adolescent fifth metacarpal neck fracture and its impact on epiphyseal growth. .... Page 7

6. Comparison of lateral entry and crossed entry pinning for pediatric supracondylar humeral fractures: a meta-analysis of randomized controlled trials. .... Page 7

7. Open total dislocation of ankle joint without fractures: A case report. .... Page 8

8. A review of the Turned-down Onto Pericapsular-tissue Hemisectioned Amputated Toe (TOPHAT) flap for wound coverage during ray amputations of the toes. .... Page 9

9. High Rates of Spacer Fracture in the Setting of Extended Trochanteric Osteotomy With a Specific Thin-Core Articulating Antibiotic Hip Spacer. .... Page 9

10. Technique and Outcomes of Hand-Assist Laparoscopic Continent Cutaneous Ileocecocystoplasty. .... Page 10

Strategy 977736/60

See full search strategy

#	Database	Search term	Results
60	Medline	((exp "METACARPAL BONES"/ OR exp "FINGER PHALANGES"/ OR exp "CARPAL BONES"/ OR exp "HAND BONES"/ OR exp METACARPUS/ OR exp FINGERS/ OR exp HAND/ OR exp "METACARPOPHALANGEAL JOINT"/ OR exp "FINGER JOINT"/ OR exp "CARPOMETACARPAL JOINTS"/ OR exp "CARPAL JOINTS"/ OR exp "HAND JOINTS"/ OR exp WRIST/ OR exp "WRIST JOINT"/) AND (exp "FINGER INJURIES"/ OR exp "HAND INJURIES"/ OR exp "WRIST INJURIES"/ OR exp "ACCIDENTAL INJURIES"/ OR exp "WOUNDS AND INJURIES"/ OR exp "WOUNDS, PENETRATING"/ OR exp "WOUNDS, NONPENETRATING"/ OR exp "VASCULAR SYSTEM INJURIES"/ OR exp "TRAUMA, NERVOUS SYSTEM"/ OR exp "TENDON INJURIES"/ OR exp "SURGICAL WOUND"/ OR exp "SOFT TISSUE INJURIES"/ OR exp REINJURIES/ OR exp "MULTIPLE TRAUMA"/ OR exp LACERATIONS/ OR exp "JOINT DISLOCATIONS"/ OR exp "HAND INJURIES"/ OR exp "FRACTURES, CARTILAGE"/ OR exp "FRACTURES, BONE"/ OR exp "FOREIGN BODIES"/ OR exp "CRUSH INJURIES"/ OR exp "ATHLETIC INJURIES"/ OR exp "ARM INJURIES"/ OR exp "AMPUTATION, TRAUMATIC"/)) AND (exp "GRAM-POSITIVE BACTERIAL INFECTIONS"/ OR exp "GRAM-NEGATIVE BACTERIAL INFECTIONS"/ OR exp "BACTERIAL INFECTIONS"/ OR exp "STAPHYLOCOCCAL INFECTIONS"/ OR exp INFECTIONS/ OR exp "WOUND INFECTION"/ OR exp TOXEMIA/ OR exp "SOFT TISSUE INFECTIONS"/ OR exp "SKIN DISEASES, INFECTIOUS"/ OR exp SEPSIS/ OR exp "BONE DISEASES, INFECTIOUS"/ OR exp "BACTERIAL INFECTIONS AND MYCOSES"/ OR exp "ARTHRITIS, INFECTIOUS"/)	1061

Contents 50 of 1061 results on Medline - ((exp "METACARPAL BONES"/ OR exp "FINGER PHALANGES"/ OR exp "CARPAL BONES"/ OR exp "HAND BONES"/ OR exp METACARPUS/ OR exp FINGERS/ OR exp HAND/ OR exp "METACARPOPHALANGEAL JOINT"/ OR exp "FINGER JOINT"/ OR exp "CARPOMETACARPAL JOINTS"/ OR exp "CARPAL JOINTS"/ OR exp "HAND JOINTS"/ OR exp WRIST/ OR exp "WRIST JOINT"/) AND (exp "FINGER INJURIES"/ OR exp "HAND INJURIES"/ OR exp "WRIST INJURIES"/ OR exp "ACCIDENTAL INJURIES"/ OR exp "WOUNDS AND INJURIES"/ OR exp "WOUNDS, PENETRATING"/ OR exp "WOUNDS, NONPENETRATING"/ OR exp "VASCULAR SYSTEM INJURIES"/ OR exp "TRAUMA, NERVOUS SYSTEM"/ OR exp "TENDON INJURIES"/ OR exp "SURGICAL WOUND"/ OR exp "SOFT TISSUE INJURIES"/ OR exp REINJURIES/ OR exp "MULTIPLE TRAUMA"/ OR exp LACERATIONS/ OR exp "JOINT DISLOCATIONS"/ OR exp "HAND INJURIES"/ OR exp "FRACTURES, CARTILAGE"/ OR exp "FRACTURES, BONE"/ OR exp "FOREIGN BODIES"/ OR exp "CRUSH INJURIES"/ OR exp "ATHLETIC INJURIES"/ OR exp "ARM INJURIES"/ OR exp "AMPUTATION, TRAUMATIC"/)) AND (exp "GRAM-POSITIVE BACTERIAL INFECTIONS"/ OR exp "GRAM-NEGATIVE BACTERIAL INFECTIONS"/ OR exp

Strategy 974100/49

See full search strategy

#	Database	Search term	Results
49	EMBASE	((exp HAND/ OR exp WRIST/ OR exp FINGER/ OR exp THUMB/) AND (exp "ACCIDENTAL INJURY"/ OR exp "BATTLE INJURY"/ OR exp "BLOOD VESSEL INJURY"/ OR exp "BLUNT TRAUMA"/ OR exp "CRUSH TRAUMA"/ OR exp "LIMB INJURY"/ OR exp "NERVOUS SYSTEM INJURY"/ OR exp "MUSCULOSKELETAL INJURY"/ OR exp "SKIN INJURY"/ OR exp "SOFT TISSUE INJURY"/ OR exp "SPORT INJURY"/ OR exp "SURGICAL INJURY"/ OR exp "TISSUE INJURY"/ OR exp "TRAUMATIC AMPUTATION"/ OR exp WOUND/ OR exp INJURY/)) AND (exp "INFECTION BURDEN"/ OR exp SUPPURATION/ OR exp "SOFT TISSUE INFECTION"/ OR exp "SKIN INFECTION"/ OR exp SEPSIS/ OR exp PUS/ OR exp "PRIMARY INFECTION"/ OR exp "PERSISTENT INFECTION"/ OR exp "MUSCULOSKELETAL INFECTION"/ OR exp "INFECTIONS ASSOCIATED WITH OTHER INFECTIONS OR CONDITIONS"/ OR exp "INFECTION RATE"/ OR exp "INFECTION COMPLICATION"/ OR exp "HOSPITAL INFECTION"/ OR exp "HEALTHCARE ASSOCIATED INFECTION"/ OR exp "HAND INFECTION"/ OR exp "GRAFT INFECTION"/ OR exp "DEVICE INFECTION"/ OR exp "BACTERIAL INFECTION"/ OR exp ABSCESS/ OR exp INFECTION/)	2551

Contents 50 of 2551 results on EMBASE - ((exp HAND/ OR exp WRIST/ OR exp FINGER/ OR exp THUMB/) AND (exp "ACCIDENTAL INJURY"/ OR exp "BATTLE INJURY"/ OR exp "BLOOD VESSEL INJURY"/ OR exp "BLUNT TRAUMA"/ OR exp "CRUSH TRAUMA"/ OR exp "LIMB INJURY"/ OR exp "NERVOUS SYSTEM INJURY"/ OR exp "MUSCULOSKELETAL INJURY"/ OR exp "SKIN INJURY"/ OR exp "SOFT TISSUE INJURY"/ OR exp "SPORT INJURY"/ OR exp "SURGICAL INJURY"/ OR exp "TISSUE INJURY"/ OR exp "TRAUMATIC AMPUTATION"/ OR exp WOUND/ OR exp INJURY/)) AND (exp "INFECTION BURDEN"/ OR exp SUPPURATION/ OR exp "SOFT TISSUE INFECTION"/ OR exp "SKIN INFECTION"/ OR exp SEPSIS/ OR exp PUS/ OR exp "PRIMARY INFECTION"/ OR exp "PERSISTENT INFECTION"/ OR exp "MUSCULOSKELETAL INFECTION"/ OR exp "INFECTIONS ASSOCIATED WITH OTHER INFECTIONS OR CONDITIONS"/ OR exp "INFECTION RATE"/ OR exp "INFECTION COMPLICATION"/ OR exp "HOSPITAL INFECTION"/ OR exp "HEALTHCARE ASSOCIATED INFECTION"/ OR exp "HAND INFECTION"/ OR exp "GRAFT INFECTION"/ OR exp "DEVICE INFECTION"/ OR exp "BACTERIAL INFECTION"/ OR exp ABSCESS/ OR exp INFECTION/)

1. 24960 Botryomycosis in an immunocompromised adult male .... Page 4
2. 25959 Clinical and histopathologic evolution of inflammatory linear verrucous epidermal nevus in a pediatric patient .... Page 4
3. 27910 A sporotrichoid rash in a landscaper and fish owner .... Page 5
4. Incident bone fracture and mortality in a large HIV cohort outpatient study, 2000-2017, USA .... Page 6

HDAS Export
Search Strategy SSI hand and wrist

26 Jan 21 - 10:47

Strategy 970556/81

See full search strategy

#	Database	Search term	Results
81	EMBASE	(infect*).ti,ab AND (((((hand*).ti,ab OR (finger*).ti,ab OR (thumb*).ti,ab OR (wrist*).ti,ab OR (phalang*).ti,ab OR (metacarpal*).ti,ab OR (carpal*).ti,ab OR (radius*).ti,ab OR (ulna*).ti,ab) AND ((nerve*).ti,ab OR (neuro*).ti,ab OR (vasc*).ti,ab OR (arter*).ti,ab OR (vessel*).ti,ab OR (tendon*).ti,ab OR (ligament*).ti,ab OR (joint*).ti,ab OR (capsul*).ti,ab OR (volar*).ti,ab OR (musc*).ti,ab)) AND ((trauma*).ti,ab OR (injur*).ti,ab OR (lacerat*).ti,ab OR (fracture*).ti,ab OR (crush*).ti,ab OR (wound*).ti,ab)) AND ((surg*).ti,ab OR (operati*).ti,ab OR (intervent*).ti,ab OR (procedur*).ti,ab OR (repair*).ti,ab OR (fix*).ti,ab OR (reconstruct*).ti,ab OR (debride*).ti,ab OR (closur*).ti,ab OR (dressing*).ti,ab))	3367

Contents 50 of 3367 results on EMBASE - (infect*).ti,ab AND (((((hand*).ti,ab OR (finger*).ti,ab OR (thumb*).ti,ab OR (wrist*).ti,ab OR (phalang*).ti,ab OR (metacarpal*).ti,ab OR (carpal*).ti,ab OR (radius*).ti,ab OR (ulna*).ti,ab) AND ((nerve*).ti,ab OR (neuro*).ti,ab OR (vasc*).ti,ab OR (arter*).ti,ab OR (vessel*).ti,ab OR (tendon*).ti,ab OR (ligament*).ti,ab OR (joint*).ti,ab OR (capsul*).ti,ab OR (volar*).ti,ab OR (musc*).ti,ab)) AND ((trauma*).ti,ab OR (injur*).ti,ab OR (lacerat*).ti,ab OR (fracture*).ti,ab OR (crush*).ti,ab OR (wound*).ti,ab)) AND ((surg*).ti,ab OR (operati*).ti,ab OR (intervent*).ti,ab OR (procedur*).ti,ab OR (repair*).ti,ab OR (fix*).ti,ab OR (reconstruct*).ti,ab OR (debride*).ti,ab OR (closur*).ti,ab OR (dressing*).ti,ab))

1. Mini-invasive Osteotomy for Pediatric Distal Radius Malunion .... Page 4

2. Allograft Interposition Bone Graft for First Metatarsal Phalangeal Arthrodesis: Salvage After Bone Loss and Shortening of the First Ray .... Page 4

3. Robotic Kidney Transplant: The Modern Era Technical Revolution .... Page 5

4. Non traumatic, single channel, low flow peroneal arteriovenous fistula leading to toe gangrene: An extremely rare observation .... Page 5

5. A Practical Approach to the Management of Digital Ulcers in Patients with Systemic Sclerosis: A Narrative Review .... Page 6

6. Top 20 Research Studies of 2020 for Primary Care Physicians .... Page 7

7. One-stage Distal Interphalangeal Joint Fusion With External Fixator for the Treatment of Septic Joint Arthritis .... Page 8

8. Vascularised fibular graft in the management of non-union of fracture shaft of radius: a less ventured entity .... Page 8

9. Upper extremity Histoplasma capsulatum treatment with isavuconazole .... Page 8

10. Long-term results after modified Burton-Pellegrini's technique in 24 cases affected by advanced rhizarthrosis .... Page 9

11. Efficacy of 2-stage revision using a prosthesis of antibiotic-loaded acrylic cement spacer with or without cortical strut allograft in infected total elbow arthroplasty .... Page 10

12. Hemiarthroplastik der Hufte uber den direkten anterioren ZugangHemiarthroplasty of the hip using the direct anterior approach .... Page 11

HDAS Export
Search Strategy SSI hand and wrist

26 Jan 21 - 10:47

Strategy 970556/242

See full search strategy

#	Database	Search term	Results
242	Medline	(infect*).ti,ab AND (((((hand*).ti,ab OR (finger*).ti,ab OR (thumb*).ti,ab OR (wrist*).ti,ab OR (phalang*).ti,ab OR (metacarpal*).ti,ab OR (carpal*).ti,ab OR (radius*).ti,ab OR (ulna*).ti,ab) AND ((nerve*).ti,ab OR (neuro*).ti,ab OR (vasc*).ti,ab OR (arter*).ti,ab OR (vessel*).ti,ab OR (tendon*).ti,ab OR (ligament*).ti,ab OR (joint*).ti,ab OR (capsul*).ti,ab OR (volar*).ti,ab OR (musc*).ti,ab)) AND ((trauma*).ti,ab OR (injur*).ti,ab OR (lacerat*).ti,ab OR (fracture*).ti,ab OR (crush*).ti,ab OR (wound*).ti,ab)) AND ((surg*).ti,ab OR (operati*).ti,ab OR (intervent*).ti,ab OR (procedur*).ti,ab OR (repair*).ti,ab OR (fix*).ti,ab OR (reconstruct*).ti,ab OR (debride*).ti,ab OR (closur*).ti,ab OR (dressing*).ti,ab))	2014

Contents 50 of 2014 results on Medline - (infect*).ti,ab AND (((((hand*).ti,ab OR (finger*).ti,ab OR (thumb*).ti,ab OR (wrist*).ti,ab OR (phalang*).ti,ab OR (metacarpal*).ti,ab OR (carpal*).ti,ab OR (radius*).ti,ab OR (ulna*).ti,ab) AND ((nerve*).ti,ab OR (neuro*).ti,ab OR (vasc*).ti,ab OR (arter*).ti,ab OR (vessel*).ti,ab OR (tendon*).ti,ab OR (ligament*).ti,ab OR (joint*).ti,ab OR (capsul*).ti,ab OR (volar*).ti,ab OR (musc*).ti,ab)) AND ((trauma*).ti,ab OR (injur*).ti,ab OR (lacerat*).ti,ab OR (fracture*).ti,ab OR (crush*).ti,ab OR (wound*).ti,ab)) AND ((surg*).ti,ab OR (operati*).ti,ab OR (intervent*).ti,ab OR (procedur*).ti,ab OR (repair*).ti,ab OR (fix*).ti,ab OR (reconstruct*).ti,ab OR (debride*).ti,ab OR (closur*).ti,ab OR (dressing*).ti,ab))

1. [Clinical effects of ulnar artery perforator chain flaps in repairing wounds on distal forearm or wrist with vascular anastomosis]. .... Page 4

2. [Wound repair and functional reconstruction of high-voltage electrical burns in wrists]. .... Page 5

3. [The pedicled groin flap for defect closure of the hand]. .... Page 5

4. Complications associated with volar locking plate fixation for distal radius fractures in 1955 cases: A multicentre retrospective study. .... Page 6

5. [Kirschner wire fixation in three joints combined with bone anchor repair for treatment of acute perilunate injury]. .... Page 7

6. Volar plate fixation for the treatment of distal radius fractures: analysis of adverse events. .... Page 8

7. [Clinical application of free peroneal perforator-based sural neurofasciocutaneous flap]. .... Page 9

8. Role of a spanning plate as an internal fixator in complex distal radius fractures. .... Page 9

9. Indications and functional outcome of the use of integra® dermal regeneration template for the management of traumatic soft tissue defects on dorsal hand, fingers and thumb. .... Page 10

10. [Vascularised Fibula and Tendon Transfer in the Comprehensive Treatment of Forearm Fracture with Gas Gangrene Complication]. .... Page 11

11. [Mini locked-plate trans-carpometacarpal joint internal fixation for treating comminuted fracture of base of the fifth metacarpal]. .... Page 11

Supplementary File 2 - Abstract Decision Tree

Step	Criteria	Decision
1	Is it a primary clinical study of human participants?	Yes – move to step 2<br><br>No – exclude
2	Is it a study of surgery for acute hand or wrist injuries?<br><br>Acute defined as surgery within 4 weeks of injury	Yes – move to step 3<br><br>No - exclude
3	Does the study report infection as an outcome?	Yes – move to step 4<br><br>No – exclude
4	Confirm that the study reports infection following surgery for acute hand/wrist injury	Yes – include, extract data<br><br>No – revisit 1-3
5	Extract the following data:<br><br>• Author<br>• Year<br>• Study design (case series, case control, cohort, RCT)<br>• Study type (retrospective or prospective)<br>• Total study participants<br>• Total surgical participants<br>• Injury site (hand, wrist, mixed)<br>• Injury type (open, closed, mixed)<br>• Treatment type (soft tissue reconstruction, external fixation, K-wire, ORIF, mixed)<br>• Number of infections in surgical participants<br>• Infection diagnosis (classification, clinical, unknown)	

Supplementary File 3. Meta-regression output

```
#Meta-regression

metareg.moderator=rma(yi, vi, data=ies.logit, mods=~Site)
metareg.moderator=rma(yi, vi, data=ies.logit, mods=~RoB)
metareg.moderator=rma(yi, vi, data=ies.logit, mods=~Intervention)
metareg.moderator=rma(yi, vi, data=ies.logit, mods=~Studytype)
metareg.moderator=rma(yi, vi, data=ies.logit, mods=~Studydesign)
metareg.moderator=rma(yi, vi, data=ies.logit, mods=~InfectionDefinition)

metareg.moderators=rma(yi, vi, data=ies.logit,

mods=~Site+Type+Intervention+RoB+Studytype+InfectionDefinition+Studydesign)

metareg.moderators$beta <- exp(metareg.moderators$beta)
metareg.moderators$ci.ub <- exp(metareg.moderators$ci.ub)
metareg.moderators$ci.lb <- exp(metareg.moderators$ci.lb)
print(metareg.moderators) #oddsratios

### Multivariate meta-regression (no test for colinearity) ###

# Mixed-Effects Model (k = 146; tau^2 estimator: REML)

# tau^2 (estimated amount of residual heterogeneity):     0.9027 (SE = 0.1489)
# tau (square root of estimated tau^2 value):             0.9501
# I^2 (residual heterogeneity / unaccounted variability): 89.49%
# H^2 (unaccounted variability / sampling variability):   9.51
# R^2 (amount of heterogeneity accounted for):            28.19%
#
# Test for Residual Heterogeneity:
#   QE(df = 129) = 1289.4455, p-val < .0001

# Test of Moderators (coefficients 2:17):
#   QM(df = 16) = 55.7220, p-val < .0001

# Model Results:

#                          OR      se     zval    pval    ci.lb   ci.ub
# intrcpt               0.4016  0.9812  -0.9297  0.3525  0.0587  2.7479
# SiteMixed             0.8499  0.3549  -0.4582  0.6468  0.4240  1.7039
# SiteWrist             0.6754  0.2434  -1.6126  0.1068  0.4192  1.0882
# TypeMixed             1.3707  0.2493   1.2651  0.2058  0.8410  2.2341
# TypeOpen              1.4903  0.2919   1.3666  0.1717  0.8410  2.6409
# InterventionKwire     0.5709  0.3654  -1.5341  0.1250  0.2789  1.1684
# InterventionMixed     0.3172  0.3345  -3.4327  0.0006  0.1647  0.6110  ***
# InterventionORIF      0.1942  0.3504  -4.6767  <.0001  0.0977  0.3860  ***
# InterventionSTR       0.3481  0.4377  -2.4105  0.0159  0.1476  0.8210    *
# RoBLow                0.9715  0.2770  -0.1045  0.9167  0.5645  1.6718
```

```
# RoBModerate                 0.7999  0.2229  -1.0017  0.3165  0.5168  1.2381
# StudytypeRetrospective        0.8312  0.2583  -0.7157  0.4742  0.5010  1.3790
# InfectionDefinitionClinical    2.1890  0.3647   2.1481  0.0317  1.0710  4.4741    *
# InfectionDefinitionUnknown     2.2493  0.4276   1.8958  0.0580  0.9729  5.1999    .
# StudydesignCase series        0.3059  0.8657  -1.3685  0.1712  0.0561  1.6687
# StudydesignCohort            0.1870  0.8579  -1.9546  0.0506  0.0348  1.0046    .
# StudydesignRCT               0.3385  0.9044  -1.1978  0.2310  0.0575  1.9923

---
#   Signif. codes:  0 ‘***’ 0.001 ‘**’ 0.01 ‘*’ 0.05 ‘.’ 0.1 ‘ ’ 1

###


vif(metareg.moderators) #colinearity

#   SiteMixed  SiteWrist  TypeMixed  TypeOpen  InterventionKwire
InterventionMixed  InterventionORIF  InterventionSTR  RoBLow  RoBModerate
StudytypeRetrospective  InfectionDefinitionClinical  InfectionDefinitionUnknown
StudydesignCase series  StudydesignCohort  StudydesignRCT
#   1.3480    1.7483    1.5671    1.9617          2.9807          2.8728          2.0623
2.6545  1.2685      1.3417               1.8834                  3.1246
2.8795               19.7996           21.9811        14.7955

### univariate meta-regression for study design (high colinearity) ###

metareg.moderators=rma(yi, vi, data=ies.logit,
                  mods=~Studydesign) #unvariate study design

metareg.moderators$beta <- exp(metareg.moderators$beta)
metareg.moderators$ci.ub <- exp(metareg.moderators$ci.ub)
metareg.moderators$ci.lb <- exp(metareg.moderators$ci.lb)
print(metareg.moderators) #oddsratios

#   Mixed-Effects Model (k = 146; tau^2 estimator: REML)

#   tau^2 (estimated amount of residual heterogeneity):     1.1878 (SE = 0.1767)
#   tau (square root of estimated tau^2 value):           1.0899
#   I^2 (residual heterogeneity / unaccounted variability): 95.48%
#   H^2 (unaccounted variability / sampling variability):   22.12
#   R^2 (amount of heterogeneity accounted for):           5.51%

#   Test for Residual Heterogeneity:
#    QE(df = 142) = 4357.1378, p-val < .0001

#   Test of Moderators (coefficients 2:4):
#     QM(df = 3) = 9.0521, p-val = 0.0286

#   Model Results:
```

```
#                          OR      se       zval    pval    ci.lb   ci.ub
#   intrcpt                 0.2582  0.8873  -1.5257  0.1271  0.0454  1.4701
#   StudydesignCase series    0.3396  0.9047  -1.1937  0.2326  0.0577  2.0002
#   StudydesignCohort        0.1929  0.8997  -1.8292  0.0674  0.0331  1.1248  .
#   StudydesignRCT           0.3039  0.9186  -1.2966  0.1948  0.0502  1.8392

#    Signif. codes:  0 '***' 0.001 '**' 0.01 '*' 0.05 '.' 0.1 ' ' 1

### multivariate meta-regression for all other variables (low colinearity) ###

metareg.moderators=rma(yi, vi, data=ies.logit,
                 mods=~Site+Type+Intervention+RoB+Studytype+InfectionDefinition)
#multivariate

metareg.moderators$beta <- exp(metareg.moderators$beta)
metareg.moderators$ci.ub <- exp(metareg.moderators$ci.ub)
metareg.moderators$ci.lb <- exp(metareg.moderators$ci.lb)
print(metareg.moderators) #oddsratios

###

# Mixed-Effects Model (k = 146; tau^2 estimator: REML)

# tau^2 (estimated amount of residual heterogeneity):     0.9715 (SE = 0.1561)
# tau (square root of estimated tau^2 value):            0.9857
# I^2 (residual heterogeneity / unaccounted variability): 90.29%
# H^2 (unaccounted variability / sampling variability):   10.30
# R^2 (amount of heterogeneity accounted for):           22.72%

# Test for Residual Heterogeneity:
#   QE(df = 132) = 1563.5878, p-val < .0001

# Test of Moderators (coefficients 2:14):
#   QM(df = 13) = 42.9688, p-val < .0001

# Model Results:

#                          OR      se       zval    pval    ci.lb   ci.ub
# intrcpt                   0.1352  0.5112  -3.9139  <.0001  0.0497  0.3683  ***
# SiteMixed                   0.7943  0.3642  -0.6324  0.5271  0.3890  1.6218
# SiteWrist                 0.7080  0.2446  -1.4116  0.1581  0.4383  1.1435
# TypeMixed                   1.1549  0.2477   0.5815  0.5609  0.7108  1.8765
# TypeOpen                    1.2735  0.2957   0.8176  0.4136  0.7133  2.2736
# InterventionKwire           0.5487  0.3723  -1.6121  0.1069  0.2645  1.1382
# InterventionMixed           0.2844  0.3352  -3.7516  0.0002  0.1474  0.5485  ***
# InterventionORIF            0.1708  0.3552  -4.9757  <.0001  0.0851  0.3426  ***
# InterventionSTR             0.3442  0.4418  -2.4141  0.0158  0.1448  0.8182   *
# RoBLow                    0.9333  0.2834  -0.2437  0.8074  0.5355  1.6264
# RoBModerate                 0.7966  0.2257  -1.0074  0.3137  0.5118  1.2398
# StudytypeRetrospective        0.8284  0.2262  -0.8325  0.4051  0.5318  1.2904
```

```
# InfectionDefinitionClinical    1.8715  0.3511   1.7850  0.0743  0.9404  3.7246   .
# InfectionDefinitionUnknown     1.8893  0.4157   1.5305  0.1259  0.8365  4.2670

# Signif. codes:  0 ‘***’ 0.001 ‘**’ 0.01 ‘*’ 0.05 ‘.’ 0.1 ‘ ’ 1

permutest(metareg.moderators) #permutation
print(metareg.moderators)

# Mixed-Effects Model (k = 146; tau^2 estimator: REML)

# tau^2 (estimated amount of residual heterogeneity):     0.9715 (SE = 0.1561)
# tau (square root of estimated tau^2 value):             0.9857
# I^2 (residual heterogeneity / unaccounted variability): 90.29%
# H^2 (unaccounted variability / sampling variability):   10.30
# R^2 (amount of heterogeneity accounted for):            22.72%

# Test for Residual Heterogeneity:
# QE(df = 132) = 1563.5878, p-val < .0001

# Test of Moderators (coefficients 2:14):
# QM(df = 13) = 42.9688, p-val < .0001

# Model Results:

#   estimate     se     zval    pval   ci.lb   ci.ub
# intrcpt                        0.1352  0.5112  -3.9139  <.0001  0.0497  0.3683  ***
# SiteMixed                      0.7943  0.3642  -0.6324  0.5271  0.3890  1.6218
# SiteWrist                      0.7080  0.2446  -1.4116  0.1581  0.4383  1.1435
# TypeMixed                      1.1549  0.2477   0.5815  0.5609  0.7108  1.8765
# TypeOpen                       1.2735  0.2957   0.8176  0.4136  0.7133  2.2736
# InterventionKwire              0.5487  0.3723  -1.6121  0.1069  0.2645  1.1382
# InterventionMixed              0.2844  0.3352  -3.7516  0.0002  0.1474  0.5485  ***
# InterventionORIF               0.1708  0.3552  -4.9757  <.0001  0.0851  0.3426  ***
# InterventionSTR                0.3442  0.4418  -2.4141  0.0158  0.1448  0.8182    *
# RoBLow                         0.9333  0.2834  -0.2437  0.8074  0.5355  1.6264
# RoBModerate                    0.7966  0.2257  -1.0074  0.3137  0.5118  1.2398
# StudytypeRetrospective         0.8284  0.2262  -0.8325  0.4051  0.5318  1.2904
# InfectionDefinitionClinical    1.8715  0.3511   1.7850  0.0743  0.9404  3.7246   .
# InfectionDefinitionUnknown     1.8893  0.4157   1.5305  0.1259  0.8365  4.2670

# Signif. codes:  0 ‘***’ 0.001 ‘**’ 0.01 ‘*’ 0.05 ‘.’ 0.1 ‘ ’ 1
```

Supplementary File 6. References for included studies

Study	Reference	Citation
Abouelela 2020	1	Abouelela A, Mubark I, Hassan M, Howells M, Ashwood N, Kitsis C. Mid-Term Outcomes of Unstable Complex Proximal Interphalangeal Joint Fracture Management Using the Ligamentotaxor® Device: A Case Series of 33 Cases. Cureus. 2020 Sep;12(9):e10519.
Abulsoud 2021	2	Abulsoud M.I., Elmarghany M., Abdelghany T., Abdelaal M., Elhalawany M.F., Zakaria A.R. A Single Intramedullary K-Wire Is Sufficient for the Management of Nonthumb Metacarpal Shaft Fractures. Adv Orthop. 2021;2021((Abulsoud, Elmarghany, Abdelghany, Abdelaal, Elhalawany) Department of Orthopedic Surgery, Faculty of Medicine, Al-Azhar University, Cairo, Egypt):9963186.
Adkison 1982	3	Adkison JW, Chapman MW. Treatment of acute lunate and perilunate dislocations. Clin Orthop. 1982 Apr;(164):199–207.
Afshar 2020	4	Afshar A, Tabrizi A, Taleb H, Safari M. Results of fracture-dislocation of interphalangeal treatment with volar buttressing hook plating techniques. Orthop Traumatol Surg Res. 2020 Jun;106(4):765–9.
Ahmad 2006	5	Ahmad M, Hussain SS, Rafiq Z, Tariq F, Khan MI, Malik SA. Management of phalangeal fractures of hand. J Ayub Med Coll Abbottabad JAMC. 2006;18(4):38–41.
Ahmad 2022	6	Ahmad F., Neral M., Hoyen H., Simcock X., Malone K. Does Time to Operative Intervention of Distal Radius Fractures Influence Outcomes? Hand N Y N. 2022;((Ahmad, Simcock) Rush University Medical Center, Chicago, United States):15589447211072220.
Aigner 2014	7	Aigner R, Debus F, Karaman Y, López-López C, Ruchholtz S, Kühne CA. [Outcomes after operative treatment of distal radius fractures - an analysis of 721 patients]. Z Orthopadie Unfallchirurgie. 2014 Aug;152(4):375–80.
Akmaz 2003	8	Akmaz I, Kiral A, Pehlivan O, Solakoğlu C. [Ligament reconstruction for the chronic instability of the traumatic thumb carpometacarpal joint]. Acta Orthop Traumatol Turc. 2003;37(3):237–43.
Al-Qattan 2005	9	Al-Qattan MM. De-epithelialized cross-finger flaps versus adipofascial turnover flaps for the reconstruction of small complex dorsal digital defects: A comparative analysis. J Hand Surg. 2005 May;30(3):549–57.
Al-Qattan 2008	10	Al-Qattan MM. The cross-digital dorsal adipofascial flap. Ann Plast Surg. 2008 Feb;60(2):150–3.
Al-Qattan 2011	11	Al-Qattan MM. Displaced unstable transverse fractures of the shaft of the proximal phalanx of the fingers in industrial workers: reduction and K-wire fixation leaving the metacarpophalangeal and proximal interphalangeal joints free. J Hand Surg Eur Vol. 2011 Sep;36(7):577–83.
Al-Qattan 2012	12	Al-Qattan MM. Saw injuries causing phalangeal neck fractures in adults. Ann Plast Surg. 2012 Jul;69(1):38–40.
Al-Shahwanii 2021	13	Al-Shahwanii Z.W., Qaryaqos S.H. Comparative study between volar locked plates versus closed reduction with percutaneous pinning in management of unstable extra-articular distal radius fractures. J Pak Med Assoc. 2021;71(12):S35–9.
Ali 2007	14	Ali H, Rafique A, Bhatti M, Ghani S, Sadiq M, Beg SA. Management of fractures of metacarpals and phalanges and associated risk factors for delayed healing. J-Pak Med Assoc. 2007;57(2):64.

An 2013	15	An Y, Wang ZX, Yuan S, Zhang YQ, Zhang J. Surgical method of fixed needle extrusiion in treatment of bony mallet finger and evaluation on curative effect. J Jilin Univ Med Ed. 2013 Mar;39(2):371–4.
Anakwe 2010	16	Anakwe R, Khan L, Cook R, McEachan J. Locked volar plating for complex distal radius fractures: Patient reported outcomes and satisfaction. J Orthop Surg. 2010 Aug;5:51.
Anderson 2004	17	Anderson JT, Lucas GL, Buhr BR. Complications of treating distal radius fractures with external fixation: a community experience. Iowa Orthop J. 2004;24:53–9.
Arora 2022	18	Arora S., Govil V., Neogi A.K., Bishnoi S., Paul S., Gupta R.K. Functional and Radiological Outcomes in Intra-Articular Fractures of Distal Radius with Volar Variable Angle Locking Plates. Trauma Mon. 2022;27(1):380–5.
Aydin 2010	19	Aydin N, Uraloglu M, Yilmaz Burhanoglu AD, Sensoz O. A prospective trial on the use of antibiotics in hand surgery. Plast Reconstr Surg. 2010 Nov;126(5):1617–23.
Azzopardi 2005	20	Azzopardi T, Ehrendorfer S, Coulton T, Abela M. Unstable extra-articular fractures of the distal radius. J Bone Jt Surg - Ser B. 2005 Jun;87(6):837–40.
Bach 2006	21	Bach HG, Gonzalez MH, Hall Jr RF. Locked Intramedullary Nailing of Metacarpal Fractures Secondary to Gunshot Wounds. J Hand Surg. 2006 Sep;31(7):1083–7.
Baldwin 2021	22	Baldwin A.J., Jackowski A., Jamal A., Vaz J., Rodrigues J.N., Tyler M., et al. Risk of surgical site infection in hand trauma, and the impact of the SARS-CoV-2 pandemic: A cohort study. J Plast Reconstr Aesthet Surg. 2021;74(11):3080–6.
Basat 2017	23	Basat NB, Allon R, Nagmi A, Wollstein R. Treatment of open fractures of the hand in the emergency department. Eur J Orthop Surg Traumatol Orthop Traumatol. 2017 Apr;27(3):415–9.
Baumgartner 2021	24	Baumgartner RE, Federer AE, Guerrero EM, Mithani SK, Ruch DS, Richard MJ. Complications of Low-Profile Plate Fixation in Metacarpal Fractures. Orthopedics. 2020 Oct;1–4.
Bauze 1999	25	Bauze A, Bain GI. Internal suture for mallet finger fracture. J Hand Surg Edinb Scotl. 1999 Dec;24(6):688–92.
Bednar 2004	26	Bednar DA, Al-Harran H. Nonbridging external fixation for fractures of the distal radius. Can J Surg. 2004 Dec;47(6):426–30.
Beeres 2021	27	Beeres FJP, Liechti R, Link BC, Babst R. Role of a spanning plate as an internal fixator in complex distal radius fractures. Oper Orthopadie Traumatol. 2020 Nov;
Bennur 2021	28	Bennur A.T., Tulaja Prasad P.V., Bennur N.A. An evaluation of outcome of surgical management of unstable comminuted fracture of distal radius using external and internal fixation. Eur J Mol Clin Med. 2021;8(4):2029–33.
Benson 2006	29	Benson LS, Edwards SL, Schiff AP, Williams CS, Visotsky JL. Dog and cat bites to the hand: treatment and cost assessment. J Hand Surg. 2006 Mar;31(3):468–73.
Berwald 2014	30	Berwald N, Khan F, Zehtabchi S. Antibiotic prophylaxis for ED patients with simple hand lacerations: a feasibility randomized controlled trial. Am J Emerg Med. 2014 Jul;32(7):768–71.
Bhat 2002	31	Bhat JA, Maajid S. Results of open proximal phalangeal fractures by ‘gantry technique’. JK Sci. 2002;4(2):83–6.
Botte 1992	32	Botte MJ, Davis JL, Rose BA, von Schroeder HP, Gellman H, Zinberg EM, et al. Complications of smooth pin fixation of fractures and dislocations in the hand and wrist. Clin Orthop. 1992 Mar;(276):194–201.
Boussakri 2014	33	Boussakri H, Elidrissi M, Azarkane M, Bensaad S, Bachiri M, Shimi M, et al. Fractures of the neck of the fifth metacarpal bone, treated by percutaneous intramedullary nailing: surgical technique, radiological and clinical results study (28 cases). Pan Afr Med J. 2014;18:187.

Brei-Thoma 2015	34	Brei-Thoma P, Vögelin E, Franz T. Plate fixation of extra-articular fractures of the proximal phalanx: do new implants cause less problems? Arch Orthop Trauma Surg. 2015 Mar;135(3):439–45.
Camacho 2021	35	Camacho E., Craviotto M., D'Oliveira L. How to Manage Complications Related to the Use of Intramedullary Screws in Metacarpal Fractures: Case Series. Rev Iberoam Cirugia Mano. 2021;49(1):4–12.
Campochiaro 2011	36	Campochiaro G, Tsatsis C, Gazzotti G, Rebuzzi M, Tronci V, Rovesta C, et al. Intra-articular distal radius fractures: A clinical and radiographic comparison of treatment with volar angular stability plate and percutaneous pinning with K-wires. J Orthop Traumatol. 2011 Oct;12.
Capo 2011	37	Capo JT, Hall M, Nourbakhsh A, Tan V, Henry P. Initial management of open hand fractures in an emergency department. Am J Orthop Belle Mead NJ. 2011 Dec;40(12):E243.
Chafik 2011	38	Chafik R, Madhar M, El Bouanani A, Saidi H, Fikry T. Le fixateur externe digital, nouveau materiel (a propos de 24 cas)The digital external fixator, new equipment (24 cases report). Chir Main. 2011 Apr;30(2):110–3.
Cheah 2012	39	Cheah AEJ, Tan DMK, Chong AKS, Chew WYC. Volar Plating for Unstable Proximal Interphalangeal Joint Dorsal Fracture-Dislocations. J Hand Surg.
Cheng 2020	40	Cheng P, Wu F, Chen H, Jiang C, Wang T, Han P, et al. Early hybrid nonbridging external fixation of unstable distal radius fractures in patients aged >=50 years. J Int Med Res. 2019;48(4).
Choubey 2022	41	Choubey R., Agarwal G., Kiradiya N., Gupta A., Jain R.K. Outcome Analysis of Fracture Lower End Radius (AO Type B & C) Treated by Orifand Plate. Eur J Mol Clin Med. 2022;9(1):1274–81.
Chul-Ho 2021	42	Chul-Ho K., Kim D.H., Han-Vit K., Kim W.J., Shin M., Kim J.W. Factors affecting healing following percutaneous intramedullary fixation of metacarpal fractures. Med U S. 2021;100(50):e27968.
Chung 2019	43	Chung KC, Malay S, Shauver MJ, Kim HM. Assessment of Distal Radius Fracture Complications Among Adults 60 Years or Older: A Secondary Analysis of the WRIST Randomized Clinical Trial. JAMA Netw Open. 2019 Jan;2(1).
Conolly 1973	44	Conolly WB, Goulston E. Problems of digital amputations: a clinical review of 260 patients and 301 amputations. Aust N Z J Surg. 1973 Sep;43(2):118–23.
Constantine 2022	45	Constantine R.S., Le E.L.H., Gehring M.B., Ohmes L., Iorio M.L. Risk Factors for Infection After Distal Radius Fracture Fixation: Analysis of Impact on Cost of Care. J Hand Surg Glob Online. 2022;4(3):123–7.
Costa 2014	46	Costa ML, Achten J, Parsons NR, Rangan A, Griffin D, Tubeuf S, et al. Percutaneous fixation with Kirschner wires versus volar locking plate fixation in adults with dorsally displaced fracture of distal radius: Randomised controlled trial. BMJ Online. 2014;349:1–10.
Costa 2022	47	Costa ML, Achten J, Ooms A, Png ME, Cook JA, Lamb SE, et al. Surgical fixation with K-wires versus casting in adults with fracture of distal radius: DRAFFT2 multicentre randomised clinical trial. BMJ. 2022 Jan 19;376:e068041.
Davies 2020	48	Davies J, Roberts T, Limb R, Mather D, Thornton D, Wade RG. Time to surgery for open hand injuries and the risk of surgical site infection: a prospective multicentre cohort study. J Hand Surg Eur Vol. 2020 Jul;45(6):622–8.
de Haseth 2015	49	de Haseth KB, Neuhaus V, Mudgal CS. Dorsal fracture-dislocations of the proximal interphalangeal joint: evaluation of closed reduction and percutaneous Kirschner wire pinning. Hand N Y N. 2015 Mar;10(1):88–93.
Dennison 2007	50	Dennison DG. Open reduction and internal locked fixation of unstable distal ulna fractures with concomitant distal radius fracture. J Hand Surg. 2007;32(6):801–5.

Dias 2020	51	Dias JJ, Brealey SD, Fairhurst C, Amirfeyz R, Bhowal B, Blewitt N, et al. Surgery versus cast immobilisation for adults with a bicortical fracture of the scaphoid waist (SWIFFT): a pragmatic, multicentre, open-label, randomised superiority trial. The Lancet. 2020 Aug;396(10248):390–401.
Donnally 2017	52	Donnally CJ, Hannay W, Rapp DA, Lekic N, Dodds SD. Machete injuries to the upper extremity. Arch Orthop Trauma Surg. 2017 Dec;137(12):1615–21.
Dumont 2007	53	Dumont C, Fuchs M, Burchhardt H, Appelt D, Bohr S, Stürmer KM. Clinical results of absorbable plates for displaced metacarpal fractures. J Hand Surg. 2007 Apr;32(4):491–6.
Duncan 1993	54	Duncan RW, Freeland AE, Jabaley ME, Meydrech EF. Open hand fractures: an analysis of the recovery of active motion and of complications. J Hand Surg. 1993 May;18(3):387–94.
Dwyer 2017	55	Dwyer CL, Crosby NE, Cooney T, Seeds W, Lubahn JD. Treating Unstable Distal Radius Fractures With a Nonspanning External Fixation Device: Comparison With Volar Locking Plates in Historical Control Group. Am J Orthop Belle Mead NJ. 46(5):E344.
Eglseder 1993	56	Eglseder WA, Hay M. Open half-pin insertion for distal radial fractures. Mil Med. 1993 Nov;158(11):708–11.
Egol 2006	57	Egol KA, Paksima N, Puopolo S, Klugman J, Hiebert R, Koval KJ. Treatment of external fixation pins about the wrist: A prospective, randomized trial. J Bone Jt Surg - Ser A. 2006 Feb;88(2):349–54.
Egol 2010	58	Egol KA, Walsh M, Romo-Cardoso S, Dorsky S, Paksima N. Distal radial fractures in the elderly: Operative compared with nonoperative treatment. J Bone Jt Surg - Ser A. 2010 Aug;92(9):1851–7.
Erken 2015	59	Erken HY, Akmaz I, Takka S, Kiral A. Reconstruction of the transverse and dorsal-oblique amputations of the distal thumb with volar cross-finger flap using the index finger. J Hand Surg Eur Vol. 2015 May;40(4):392–400.
Fan 2016	60	Fan J, Jiang B, Yuan F, Li SZ, Zhou JQ, Mei J, et al. [Clinical effect of compound internal fixations in treating extreme distal radial fractures]. Zhonghua Wai Ke Za Zhi. 2016 Oct;54(10):766–71.
Faruqui 2012	61	Faruqui S, Stern PJ. Percutaneous pinning of proximal phalangeal base fractures: Unacceptable outcomes: Level 3 evidence. J Hand Surg. 2012 Sep;37(8):38–9.
Feldman 2021	62	Feldman G., Orbach H., Rozen N., Rubin G. Usefulness of prophylactic antibiotics in preventing infection after internal fixation of closed hand fractures. Hand Surg Rehabil. 2021;40(2):167–70.
Ficke 2018	63	Ficke B, Ransom EF, Hess MC, Moon AS, McKissack HM, Shah A, et al. Outcomes of Staged Treatment for Complex Distal Radius Fractures. Cureus. 2018 Sep;10(9):e3273.
Foldhazy 2010	64	Földhazy Z, Ahrengart L. External fixation versus closed treatment of displaced distal radial fractures in elderly patients: a randomized controlled trial. Curr Orthop Pract. 2010 Jun;21(3):288.
Frueh 2014	65	Frueh FS, Kunz VS, Gravestock IJ, Held L, Haefeli M, Giovanoli P, et al. Primary flexor tendon repair in zones 1 and 2: early passive mobilization versus controlled active motion. J Hand Surg. 2014 Jul;39(7):1344–50.
Fu 2006	66	Fu YC, Chien SH, Huang PJ, Chen SK, Tien YC, Lin GT, et al. Use of an external fixation combined with the buttress-maintain pinning method in treating comminuted distal radius fractures in osteoporotic patients. J Trauma - Inj Infect Crit Care. 2006 Feb;60(2):330–3.
Galivanche 2021	67	Galivanche A.R., FitzPatrick S., Dussik C., Malpani R., Nduaguba A., Varthi A.G., et al. A Matched Comparison of Postoperative Complications Between Smokers and Nonsmokers Following Open Reduction Internal Fixation of Distal Radius Fractures. J Hand Surg. 2021;46(1):1.

Gereli 2010	68	Gereli A, Nalbantoğlu U, Kocaoğlu B, Türkmen M. Comparison of palmar locking plate and K-wire augmented external fixation for intra-articular and comminuted distal radius fractures. Acta Orthop Traumatol Turc. 2010;44(3):212–9.
Ghosh 2013	69	Ghosh S, Sinha RK, Datta S, Chaudhuri A, Dey C, Singh A. A study of hand injury and emergency management in a developing country. Int J Crit Illn Inj Sci. 2013 Oct;3(4):229–34.
Goslings 1999	70	Goslings JC, Boxma H, Hauet EJ, Van Riet YEA, Keeman JN, Broekhuizen AH. Three-dimensional dynamic external fixation of distal radial fractures. A prospective study. Injury. 1999 May;30(6):421–30.
Gradl 2005	71	Gradl G, Gierer P, Mittlmeier T, Jupiter JB. Fractures of the distal radius treated with a nonbridging external fixation technique using multiplanar K-wires. J Hand Surg. 2005 Sep;30(5):960–8.
Grossman 1981	72	Grossman JAI, Adams JP, Kunec J. Prophylactic antibiotics in simple hand lacerations. J Am Med Assoc. 1981;245(10):1055–6.
Grundberg 1981	73	Grundberg AB. Intramedullary fixation for fractures of the hand. J Hand Surg. 1981 Nov;6(6):568–73.
Guerrero 2019	74	Guerrero EM, Baumgartner RE, Federer AE, Mithani SK, Ruch DS, Richard MJ. Complications of Low-Profile Plate Fixation of Phalanx Fractures. Hand N Y N. 2019 Jun;
Gumussuyu 2021	75	Gumussuyu G., Asoglu M.M., Guler O., May H., Turan A., Kose O. Extension pin block technique versus extension orthosis for acute bony mallet finger; a retrospective comparison. Orthop Traumatol Surg Res. 2021;107(5):102764.
Hargreaves 2004	76	Hargreaves DG DS. Kirschner wire pin tract infection rates: a randomized controlled trial between percutaneous and buried wires. J Hand Surg. 2004;29(4):374–6.
Henry 2008	77	Henry M. Specific complications associated with different types of intrinsic pedicle flaps of the hand. J Reconstr Microsurg. 2008 Apr;24(3):221–5.
Henry 2020	78	Henry TW, Matzon JL, McEntee RM, Lutsky KF. Outcomes of Type I Open Distal Radius Fractures: A Comparison of Delayed and Urgent Open Reduction Internal Fixation. Hand N Y N. 2020 Nov;1558944720964965.
Hery 2022	79	Hery J.-C., Champain G., Lombard A., Hulet C., Malherbe M. Relevance of antibiotic prophylaxis in the management of surgical emergency open hand trauma. Hand Surg Rehabil. 2022;41(1):137–41.
Horton 2003	80	Horton TC, Hatton M, Davis TRC. A prospective randomized controlled study of fixation of long oblique and spiral shaft fractures of the proximal phalanx: Closed reduction and percutaneous Kirschner wiring versus open reduction and lag screw fixation. J Hand Surg. 2003 Feb;(1):5–9.
Hove 1997	81	Hove LM, Furnes O, Nilsen PT, Solheim E, Molster AO, Oulie HE. Closed reduction and external fixation of unstable fractures of the distal radius. Scand J Plast Reconstr Surg Hand Surg. 1997;31(2):159–64.
Hove 2010	82	Hove LM, Krukhaug Y, Helland P, Revheim K, Finsen V. Dynamic compared with static external fixation of unstable fractures of the distal part of the radius: A prospective, randomized multicenter study. J Bone Jt Surg - Ser A. 2010 Jul;92(8):1687–96.
Huang 2018	83	Huang YC, Hsu CJ, Renn JH, Lin KC, Yang SW, Tarng YW, et al. WALANT for distal radius fracture: open reduction with plating fixation via wide-awake local anesthesia with no tourniquet. J Orthop Surg. 2018 Aug;13(1):195.
Hussain 2013	84	Hussain MA, Mui S, Pandya A, Tan E, Pandya AN. Pulp tissue anchor repair for the zone i flexor tendon injury: Introduction of a new and cost-effective technique. Eur J Plast Surg. 2013 Jan;36(1):27–30.
Ibrahim 2022	85	Ibrahim J., Hoffman R.A., Silva S., Criner-Woozley K. Incidence of Nerve Transection in Upper Extremity Gunshot Wounds. Bull Hosp Joint Dis. 2022;80(2):224–7.

Ignacio 1999	86	Ignacio H, Chueire AG, Caravalho Filho G, De Oliveira Carneiro M. Fixacao externa radio-radial no tratamento de fratura da extremidade distal do radioExternal radio-radial fixation in the treatment of distal end radius fractures. Rev Bras Ortop. 1999;34(9):535–42.
Johandi 2017	87	Johandi F, Sechachalam S. Clinical and functional outcome of open primary repair of triangular fibrocartilage complex tears associated with distal radius fractures. J Orthop Surg Hong Kong. 2017 Jan;25(1):2309499017690984.
Johnson 2021	88	Johnson A.R., Lainez S.S., Santos H.J., Chen A.D., Lainez C., Lin S.J., et al. Beyond the Tip of the Blade: An Investigation of Upper Extremity Machete Injuries in Honduras. J Reconstr Microsurg. 2021;37(3):263–71.
Jokhio 2021	89	Jokhio M.F., Rehman N.U., Baloch R.A., Soomro M.A., Silro A.K., Keerio N.H., et al. Open reduction and internal fixation of intra-articular distal radius fracture by buttress plate: An outcome assessment. Int J Res Pharm Sci. 2021;12(3):1939–42.
Joo 2022	90	Joo M.S., Kang H.J., Yu H.K., Lee J.S. Outcomes of Primary Volar Locking Plate Fixation of Open Distal Radius Fractures. J Hand Surg Asian-Pac Vol [Internet]. 2022;((Joo, Kang, Yu) Department Of Orthopedic Surgery, Wonkwang University Hospital, Iksan, South Korea). Available from: https://www.worldscientific.com/worldscinet/jhs
Joosten 1999	91	Joosten U, Joist A, Frebel T, Rieger H. [The treatment of unstable fractures of the distal radius using a bridging external fixator. Results from a long-term evaluation]. Chir Z Alle Geb Oper Medizen. 1999 Nov;70(11):1315–22.
Jose 2017	92	Jose A, Suranigi SM, Deniese PN, Babu AT, Rengasamy K, Najimudeen S. Unstable Distal Radius Fractures Treated by Volar Locking Anatomical Plates. J Clin Diagn Res JCDR. 2017 Jan;11(1):RC04.
Kadhum 2022	93	Kadhum M., Georgiou A., Kanapathy M., Reissis D., Akhavani M., Burr N., et al. Operative outcomes for wide awake local anesthesia versus regional and general anesthesia for flexor tendon repair. Hand Surg Rehabil. 2022;41(1):125–30.
Khan 2022	94	Khan H, Adil A, Ul Ain N, Qureshi BA, Chishti UF, Malik TS. Outcome of Buried Versus Exposed Kirchner Wires in Terms of Infection in Fractures of Phalanges and Metacarpal Bones of Hand. Cureus. 2022;14(2):e22515.
Kilic 2015	95	Kilic B, Zekioğlu A, Yücel AS. Flexor Tendon Injuries and Treatment Results of 67 Patients. Pak J Biol Sci PJBS. 2015 Jan;18(1):32–6.
Kim 2013	96	Kim JK, Park SD. Outcomes after volar plate fixation of low-grade open and closed distal radius fractures are similar. Clin Orthop. 2013 Jun;471(6):2030–5.
Kivi 2011	97	Mardani Kivi M, Asadi K, Hashemi Motlagh K, Shakiba M. Distal radius fracture, a comparison between closed reduction and long arm cast Vs. Closed reduction and percutaneous pinning and short arm cast. Shiraz E Med J. 2011 Jul;12(3):155–61.
Kömürcü 2005	98	Komurcu M, Kamaci L, Ozdemir MT, Atesalp AS, Basbozkurt M. Radius distal uc kiriklarinin (AO tip C2-C3) eksternal fiksatorle tedavisiTreatment of AO type C2-C3 fractures of the distal end of the radius with external fixation. Acta Orthop Traumatol Turc. 2005;39(1):39–45.
Kucukguven 2019	99	Kucukguven A, Uzun H, Menku FD, Sert G, Aksu AE. Endoscopic retrieval of retracted flexor tendons: An atraumatic technique. J Plast Reconstr Aesthetic Surg JPRAS. 2019 Apr;72(4):622–7.
Kural 2020	100	Kural C, Tanriverdi B, Erçin E, Baca E, Yilmaz M. The surgical outcomes of trans-scaphoid perilunate fracture-dislocations. Turk J Med Sci. 2020 Feb;50(1):25–30.
Kurzen 2006	101	Kurzen P, Fusetti C, Bonaccio M, Nagy L. Complications after plate fixation of phalangeal fractures. J Trauma. 2006 Apr;60(4):841–3.
Lallemand 2002	102	Lallemand S, de Jesse Levas A. [Elastic stable intramedullary nailing of hand bones. ECMES even with the hand]. Chir Main. 2002 May;21(3):176–81.

Langridge 2021	103	Langridge B, Griffin M, Akhavani M, Butler PE. Bennett's Fracture Repair-Which Method Results in the Best Functional Outcome? A Retrospective Cohort Analysis and Systematic Literature Review of Patient-Reported Functional Outcomes. J Hand Microsurg. 2021;13(2):81–8.
Lassner 2001	104	Lassner F, Becker M, Pallua N. [Therapy of soft tissue trauma in the acute phase of severe hand injuries]. Chir Z Alle Geb Oper Medizen. 2001 Dec;72(12):1439–45.
Lattman 2011	105	Lattmann T, Meier C, Dietrich M, Forberger J, Platz A. Results of volar locking plate osteosynthesis for distal radial fractures. J Trauma - Inj Infect Crit Care. 2011 Jun;70(6):1510–8.
Lauder 2015	106	Lauder A, Allan CH, Hanel DP, Huang JI, Agnew S, Bakri K. Functional Outcomes Following Bridge Plate Fixation for Distal Radius Fractures. J Hand Surg. 2015 Aug;40(8):1554–62.
Lawson 2021	107	Lawson A., Naylor J.M., Buchbinder R., Ivers R., Balogh Z.J., Smith P., et al. Surgical Plating vs Closed Reduction for Fractures in the Distal Radius in Older Patients: A Randomized Clinical Trial. JAMA Surg. 2021;156(3):229–37.
Lee 2003	108	Lee HC, Wong YS, Chan BK, Low CO. Fixation of distal radius fractures using AO titanium volar distal radius plate. Hand Surg Int J Devoted Hand Up Limb Surg Relat Res J Asia-Pac Fed Soc Surg Hand. 2003 Jul;8(1):7–15.
Lee 2020	109	Lee JH, Lee JK, Park JS, Kim DH, Baek JH, Kim YJ, et al. Complications associated with volar locking plate fixation for distal radius fractures in 1955 cases: A multicentre retrospective study. Int Orthop. 2020 Oct;44(10):2057–67.
Lee 2022	110	Lee Y.J., Ryoo H.J., Shim H.-S. Prevention of postoperative adhesions after flexor tendon repair with acellular dermal matrix in Zones III, IV, and V of the hand A randomized controlled (CONSORT-compliant) trial. Med U S. 2022;101(3):e28630.
Levy 2021	111	Levy K.H., Sedaghatpour D., Avoricani A., Kurtzman J.S., Koehler S.M. Outcomes of an aseptic technique for Kirschner wire percutaneous pinning in the hand and wrist. Injury. 2021;52(4):889–93.
Li 2022	112	Li Y., Zhang H. Effect of Joint Use of External Minifixator and Titanium Lockplate on Total Active Motion Range and Hand Function Recovery in Comminuted Metacarpal and Phalanx Fracture Patients. J Healthc Eng. 2022;2022((Li, Zhang) Department of Orthopeadic Surgery, The First Affiliated Hospital of Jiamusi University, Heilongjiang, Jiamusi 154003, China):3566364.
Li C 2016	113	Li C, Xu X, Su Y, Zhou T, Fan X, Xu Y. Treatment of scaphoid fractures using a memory alloy nail-feet-fixation device. ANZ J Surg. 2016 Jul;86(7–8):584–8.
Li S 2016	114	Li S, Wu K, Xie L, Chen L. [MODIFIED INTRAMEDULLARY FIXATION WITH TWO Kirschner WIRES FOR EXTRA-ARTICULAR FRACTURE OF PROXIMAL PHALANGEAL BASE]. Zhongguo Xiu Fu Chong Jian Wai Ke Za Zhi Zhongguo Xiufu Chongjian Waike Zazhi Chin J Reparative Reconstr Surg. 2016 Aug;30(8):935–8.
Liechti 2022	115	Liechti R., Babst R., Hug U., Link B.-C., van de Wall B.J.M., Knobe M., et al. The spanning plate as an internal fixator in complex distal radius fractures: a prospective cohort study. Eur J Trauma Emerg Surg Off Publ Eur Trauma Soc. 2022;48(3):2369–77.
Liu Q 2021	116	Liu Q, Guo W, Qu W, Ou X, Li R, Tian H. Treatment of volar defects of the finger using dorsal digital-metacarpal flap versus free medial plantar artery flap: a comparative study. BMC Surg. 2021 Jan;21(1):52.
Liu Y 2020	117	Liu Y, Bai YM. Efficacy of non-bridging external fixation in treating distal radius fractures. Orthop Surg. 2020 Jun;12(3):776–83.
Lopes 1997	118	Lopes EI, Chakkour I, Lopes Filho JD, Gomes MD, Da Costa AC, Apocalypse MS. Tratamento das fraturas do radio distal: Uso da placa em 'T' volarTreatment of fractures of distal radius: Use of 'T' plate. Rev Bras Ortop. 1997;32(3):189–200.
Lundqvist 2022	119	Lundqvist E, Fischer P, Wretenberg P, Pettersson K, Lopez Personat A, Sagerfors M. Volar Locking Plate Compared With Combined Plating of AO Type C Distal Radius Fractures: A Randomized Controlled Study of 150 Cases. J Hand Surg. 2022 Sep 1;47(9):813–22.

Lutsky 2019	120	Lutsky K, Edelman D, Leinberry C, Takei TR, Kwok M, Gallant G, et al. A Prospective Evaluation of Complications after Use of Exposed Pins in the Hand and Wrist. Plast Reconstr Surg. 2019 Jun;
Lutz 2014	121	Lutz K, Yeoh KM, MacDermid JC, Symonette C, Grewal R. Complications associated with operative versus nonsurgical treatment of distal radius fractures in patients aged 65 years and older. J Hand Surg. 2014 Jul;39(7):1280–6.
Mahmood 2022	122	Mahmood B., Golub I.J., Ashraf A.M., Ng M.K., Vakharia R.M., Choueka J. Risk Factors for Infections Following Open Reduction and Internal Fixation for Distal Radius Fractures An Analysis of the Medicare Claims Database. Bull Hosp Joint Dis. 2022;80(2):228–33.
Maradei-Pereira 2021	123	Maradei-Pereira JAR, Dos Santos AP, Martins JR, Maradei-Pereira MR. Infection after buried or exposed K-wire fixation of distal radial fractures: a randomized clinical trial. J Hand Surg Eur Vol. 2020 Jul;
Marshall 1978	124	Marshall KA, Wolfort FG, Edlich RF. Immediate insertion of silicone rubber rods in fingers with cut flexor tendons. Plast Reconstr Surg. 1978 Jan;61(1):77–9.
Marsland 2012	125	Marsland D, Sanghrajka AP, Goldie B. Static monolateral external fixation for the Rolando fracture: a simple solution for a complex fracture. Ann R Coll Surg Engl. 2012 Mar;94(2):112–5.
Martins 2021	126	Martins A., Artuso M., Marc Claise J. Bipedicle strap flaps for reconstruction of longitudinal dorsal finger defects: a review of 42 cases. J Hand Surg Eur Vol. 2021;46(8):873–6.
Mattiassich 2013	127	Mattiassich G, Mayrhofer-Stelzhammer M, Huber W, Dorninger L, Kröpfl A. [Early functional treatment of intra-articular fractures of the proximal interphalangeal joint of the finger using a modified traction device]. Handchir Mikrochir Plast Chir Organ Deutschsprachigen Arbeitsgemeinschaft Handchir Organ Deutschsprachigen Arbeitsgemeinschaft Mikrochir Peripher Nerven Gefasse Organ V. 2013 Jun;45(3):167–74.
Matullo 2015	128	Matullo KS, Dennison DG. Outcome following distally locked volar plating for distal radius fractures with metadiaphyseal involvement. Hand N Y N. 2015 Jun;10(2):292–6.
McCallister 2006	129	McCallister WV, Ambrose HC, Katolik LI, Trumble TE. Comparison of pullout button versus suture anchor for zone I flexor tendon repair. J Hand Surg. 2006 Feb;31(2):246–51.
Meaike 2021	130	Meaike J.J., Athens C., Sgromolo N., Shin A.Y., Rhee P.C. Wound Complications with Multiple Wrist Incisions in Distal Radius Reconstruction. J Wrist Surg [Internet]. 2021;((Meaike, Athens, Shin, Rhee) Department of Orthopedic Surgery, Mayo Clinic, 200 First Street SW, Rochester, MN 55905, United States). Available from: https://www.thieme-connect.com/products/ejournals/journal/10.1055/s-00000183
Meena 2021	131	Meena M., Ghosliya R.P., Naveen S., Tatwal D.K. Study of Functional Outcome of Intra-Articular Distal end Radius Fractures in Adults Treated by Distal Radius Locking Plate. Eur J Mol Clin Med. 2021;8(4):2526–34.
Mehdinasab 2012	132	Mehdinasab SA, Pipelzadeh MR, Sarrafan N. Results of primary extensor tendon repair of the hand with respect to the zone of injury. Arch Trauma Res. 2012;1(3):131–4.
Miao 2015	133	Miao D, Yang G, Zhang L, Wu J. Case control study on therapeutic effects of dynamic external fixtor combined with limited internal fixation and cross K-wires fixation for the treatment of Pilon fractures of the proximal interphalangeal joint. Zhongguo Gu Shang China J Orthop Traumatol. 2015 Oct;28(10):920–3.
Michael 2022	134	Michael G, George K, Canjirathinkal MA, Ratna P, Francis J. Functional Outcome of Joshi’s External Stabilization System Fixation in Distal Radius Fractures. Cureus. 2022;14(4):e24215.
Minhas 2019	135	Minhas SV, Catalano LW. Comparison of Open and Closed Hand Fractures and the Effect of Urgent Operative Intervention. J Hand Surg. 2019 Jan;44(1):65.

Mishra 2021	136	Mishra R.K., Sharma B.P., Kumar A., Sherawat R. A comparative study of variable angle volar plate and bridging external fixator with K-wire augmentation in comminuted distal radius fractures. Chin J Traumatol - Engl Ed. 2021;24(5):301–5.
Naguib 2022	137	Naguib M., Ramadan M., Ali T., El-Tantawy A. Simplified Kirschner-wire-based dynamic external fixator for unstable proximal interphalangeal joint fractures. Eur J Trauma Emerg Surg Off Publ Eur Trauma Soc. 2022;48(1):71–9.
Nazerani 2009	138	Nazerani S, Motamedi MHK. Ectopic single-finger transplantation, a novel technique for nonreplantable digits: assessment of 24 cases--presenting the 'piggyback' method. Tech Hand Up Extrem Surg. 2009 Jun;13(2):65–74.
Nazerani 2011	139	Nazerani S, Motamedi MHK, Ebadi MR, Nazerani T, Bidarmaghz B. Experience with distal finger replantation: a 20-year retrospective study from a major trauma center. Tech Hand Up Extrem Surg. 2011 Sep;15(3):144–50.
Nguyen 2018	140	Nguyen DT, Tran TS, Nguyen BH, Pham VD. [Covering of Finger-Tissue Loss by Local Flaps in Hanoi, Vietnam]. Bull Soc Pathol Exot 1990. 2018;111(2):121–5.
Noor 2021	141	Noor F., Nasir H.M., Zahra F., Kumar J., Ali S. Compare the outcomes of mini-plate versus K-wire fixation in patients with shaft of metacarpal fractures. Pak J Med Health Sci. 2021;15(6):1469–71.
Nygaard 2011	142	Nygaard M, Dahlin LB. Dog bite injuries to the hand. J Plast Surg Hand Surg. 2011 Apr;45(2):96–101.
Ochman 2006	143	Ochman S, Frerichmann U, Armsen N, Raschke MJ, Meffert RH. [Is use of the fixateur externe no longer indicated for the treatment of unstable radial fracture in the elderly?]. Unfallchirurg. 2006 Dec;109(12):1050–7.
Ornelli 2019	144	Ornelli M, Ruocco G, Kaciulyte J, Lazzaro L, Felici N. Immediate vs. delayed toe-to-thumb transfer: Is the infection rate greater? Handchir Mikrochir Plast Chir Organ Deutschsprachigen Arbeitsgemeinschaft Handchir Organ Deutschsprachigen Arbeitsgemeinschaft Mikrochir Peripher Nerven Gefasse Organ V. 2019 Dec;51(6):434–9.
Page 1998	145	Page SM, Stern PJ. Complications and range of motion following plate fixation of metacarpal and phalangeal fractures. J Hand Surg. 1998 Sep;23(5):827–32.
Pan 2020	146	Pan J, Li M, Huang Y, Dong J, Wang X, Wang L. Pure perforator free sensory proximal ulnar artery perforator flap for resurfacing hand defects. J Int Med Res. 2020 May;48(5):300060520922396.
Parmaksizoglu 2016	147	Parmaksizoglu AS, Ozkaya U, Mutlu H, Cetin U, Bilgili F. Fixation of extra-articular distal humeral fractures with a lateral approach and a locked plate: an alternative method. Acta Orthop Traumatol Turc. 2016;50(2):132–8.
Peacock 1988	148	Peacock KC, Hanna DP, Kirkpatrick K, Breidenbach WC, Lister GD, Firrell J. Efficacy of perioperative cefamandole with postoperative cephalexin in the primary outpatient treatment of open wounds of the hand. J Hand Surg. 1988 Nov;13(6):960–4.
Pelissier 2015	149	Pélissier P, Gobel F, Choughri H, Alet JM. Proximal interphalangeal joint fractures treated with a dynamic external fixator: A multicenter and retrospective study of 88 cases. Chir Main. 2015 Oct;34(5):245–50.
Platt 1995	150	Platt AJ PR. Post-operative infection following hand surgery: Guidelines for antibiotic use. J Hand Surg Eur Vol. 1995;20(5):685–90.
Prunieres 2016	151	Prunieres G, Gouzou S, Facca S, Matheron AS, Maire N, Hidalgo Diaz JJ, et al. Traitement des fractures instables de la phalange distale par brochage extra-articulaire de l'interphalangienne distale : a propos de 12 casTreatment of unstable distal phalanx fractures by extra-articular DIP pinning: A series of 12 cases. Hand Surg Rehabil. 2016 Oct;35(5):330–4.
Rabarin 2016	152	Rabarin F, Saint Cast Y, Jeudy J, Fouque PA, Cesari B, Bigorre N, et al. Cross-finger flap for reconstruction of fingertip amputations: Long-term results. Orthop Traumatol Surg Res OTSR. 2016 Jun;102(4).
Rafique 2006	153	Rafique A, Ghani S, Sadiq M, Siddiqui IA. Kirschner wire pin tract infection rates between percutaneous and buried wires in treating metacarpal and phalangeal fractures. J Coll Physicians Surg--Pak JCPSP. 2006 Aug;16(8):518–20.

Rajappa 2014	154	Rajappa S, Menon PG, Kumar MM, Raj DG. Early active motion protocol following triple Kessler repair for flexor tendon injury. J Orthop Surg Hong Kong. 2014 Apr;22(1):96–9.
Rao 2022	155	Rao V., Akiki R.K., Crozier J.W., Bhatt R.A., Schmidt S.T., Kalliainen L.K. Rethinking the Need for Nail Plate Removal: A Comparison of the Risks Between Standard Nail Bed Repair and Nonoperative Management. Ann Plast Surg. 2022;88(3 Supplement 3):S209–13.
Raza 2021	156	Raza A., Saleem H.M.K., Chaudhry M., Khalid M.U. Radiological and functional outcome of distal radius fracture treated conservatively (mua plaster of paris) vs percutaneous k-wire fixation. Pak J Med Health Sci. 2021;15(8):1842–5.
Ren 2021	157	Ren J, Lu L, Gao F. The use of the posterior interosseous artery flap and anterolateral thigh flap for post-traumatic soft tissue reconstruction of the hand. Medicine (Baltimore). 2021 Jul 2;100(26):e26517.
Rendon-Medina 2022	158	Rendón-Medina MA, Hanson-Viana E, Mendoza-Vélez M de los Á, Vargas-Rocha JM, Rojas-Ortiz JA, Hernandez-Ordoñez R, et al. Comparison of wire versus Nylon in Bonny-Mallet Finger treated with pull-out surgery. Cir Cir. 2022 Dec 1;90(92):023–8.
Reissner 2012	159	Reissner L, Gienck M, Weishaupt D, Platz A, Kilgus M. [Clinical and radiological results after operative treatment of mallet fracture using Kirschner wire technique]. Handchir Mikrochir Plast Chir Organ Deutschsprachigen Arbeitsgemeinschaft Handchir Organ Deutschsprachigen Arbeitsgemeinschaft Mikrochir Peripher Nerven Gefasse Organ V. 2012 Jan;44(1):11–6.
Ridley 2017	160	Ridley TJ, Freking W, Erickson LO, Ward CM. Incidence of Treatment for Infection of Buried Versus Exposed Kirschner Wires in Phalangeal, Metacarpal, and Distal Radial Fractures. J Hand Surg. 2017 Jul;42(7):525–31.
Ring 1997	161	Ring D, Jupiter JB, Brennwald J, Buchler U. Prospective multicenter trial of a plate for dorsal fixation of distal radius fractures. J Hand Surg. 1997;22(5):777–84.
Rocchi 2006	162	Rocchi L, Genitiempo M, Fanfani F. Percutaneous fixation of mallet fractures by the 'umbrella handle' technique. J Hand Surg Edinb Scotl. 2006 Aug;31(4):407–12.
Saied 2019	163	Saied AR, Sabet Jahromi M. Treatment of proximal phalanx fractures: transarticular pinning the metacarpophalangeal joint or cross pinning from the base of the proximal phalanx-a prospective study. Eur J Trauma Emerg Surg Off Publ Eur Trauma Soc. 2019 Aug;45(4):737–43.
Saint-Cyr 2006	164	Saint-Cyr M, Miranda D, Gonzalez R, Gupta A. Immediate corticocancellous bone autografting in segmental bone defects of the hand. J Hand Surg Edinb Scotl. 2006 Apr;31(2):168–77.
Shakoor 2021	165	Shakoor N., Ahmed U., Nawazish U.A., Ansari H.R., Saddiq S., Aziz A. Functional outcome of closed metacarpal fractures treated with mini fragment plates and screws. Pak J Med Health Sci. 2021;15(10):2609–10.
Shetty 1997	166	Shetty PC, Dicksheet S, Scalea TM. Emergency department repair of hand lacerations using absorbable vicryl sutures. J Emerg Med. 1997;15(5):673–4.
Sim 2021	167	Sim WP, Ng HJH, Liang BZ, Rajaratnam V. Can Open Hand Injuries Wait for Their Surgery in a Tertiary Hospital?. J Hand Microsurg. 2021;13(3):157–63.
Singh 2019	168	Singh T, Jayawardhana R, Craigen M, Rajaratnam V. Volar Buttress Plating for Unstable Dorsal Fracture-Dislocations of the Proximal Interphalangeal Joint. J Hand Microsurg. 2019 Aug;11(2):106–10.
Skochdopole 2022	169	Skochdopole A., Tarabishy S., Hermiz S., Mailey B., Herrera F.A. Open Reduction Internal Fixation of Distal Radius Fractures: Retrospective Cohort Analysis of the Geriatric Population Using the NSQIP Database. Hand N Y N. 2022;17(2):319–25.
Smith 1988	170	Smith RS, Crick JC, Alonso J, Horowitz M. Open reduction and internal fixation of volar lip fractures of the distal radius. J Orthop Trauma. 1988;2(3):181–7.

Solari 2021	171	Solari M, Kapur B, Benjamin-Laing H, Klass BR, Cheung G, Brown DJ. Reducing the incidence of pin site infection in hand surgery with the use of a protocol from Ilizarov. J Hand Surg Eur Vol. 2021 Jun;46(5):482–7.
Stahl 2001	172	Stahl S, Schwartz O. Complications of K-wire fixation of fractures and dislocations in the hand and wrist. Arch Orthop Trauma Surg. 2001 Oct;121(9):527–30.
Starker 1995	173	Starker I, Eaton RG. Kirschner wire placement in the emergency room. Is there a risk? J Hand Surg Edinb Scotl. 1995 Aug;20(4):535–8.
Starr 2021	174	Starr B.W., Dembinski D.R., Yuan F., Lax E.A., Yalamanchili S., Megee D.M. Point Blank: A Retrospective Review of Self-inflicted Gunshot Wounds to the Hand. Hand N Y N. 2021;((Starr, Dembinski, Yuan, Lax, Yalamanchili, Megee) University of Cincinnati College of Medicine, OH, United States):15589447211014604.
Stone 1998	175	Stone JF, Davidson JS. The role of antibiotics and timing of repair in flexor tendon injuries of the hand. Ann Plast Surg. 1998 Jan;40(1):7–13.
Supichyangur 2022	176	Supichyangur K., Tananon T., Sripakdee S.-A., Chunyawongsak V. Prospective Comparison of the Early Outcomes of Headless Compression Screw and Percutaneous K-Wire Fixation in Metacarpal Fractures. J Hand Surg [Internet]. 2022;((Supichyangur, Tananon, Sripakdee, Chunyawongsak) Hand and Reconstructive Microsurgery Unit, Department of Orthopedic Surgery, Rajavithi Hospital, College of Medicine, Rangsit University, Bangkok, Thailand). Available from: http://www.elsevier.com/inca/publications/store/6/2/3/1/4/5/index.htt
Suprock 1990	177	Suprock MD, Hood JM, Lubahn JD. Role of antibiotics in open fractures of the finger. J Hand Surg. 1990 Sep;15(5):761–4.
Swanson 1991	178	Swanson TV, Szabo RM, Anderson DD. Open hand fractures: prognosis and classification. J Hand Surg. 1991 Jan;16(1):101–7.
Szalay 2011	179	Szalay G, Schleicher I, Kraus R, Pavlidis T, Schnettler R. [Operative treatment of the mallet fracture using a hook plate]. Handchir Mikrochir Plast Chir Organ Deutschsprachigen Arbeitsgemeinschaft Handchir Organ Deutschsprachigen Arbeitsgemeinschaft Mikrochir Peripher Nerven Gefasse Organ V. 2011 Feb;43(1):46–53.
Tarallo 2013	180	Tarallo L, Mugnai R, Zambianchi F, Adani R, Catani F. Volar plate fixation for the treatment of distal radius fractures: analysis of adverse events. J Orthop Trauma. 2013 Dec;27(12):740–5.
Tarallo 2020	181	Tarallo L, Giorgini A, Novi M, Zambianchi F, Porcellini G, Catani F. Volar PEEK plate for distal radius fracture: analysis of adverse events. Eur J Orthop Surg Traumatol Orthop Traumatol. 2020 Oct;30(7):1293–8.
Tareen 2019	182	Tareen J, Kaufman AM, Pensy RA, O'Toole RV, Eglseder WA. Timing of treatment of open fractures of the distal radius in patients younger than 65 years. Orthopedics. 2019;42(4):219–25.
Terndrup 2018	183	Terndrup M, Jensen T, Kring S, Lindberg-Larsen M. Should we bury K-wires after metacarpal and phalangeal fracture osteosynthesis? Injury. 2018 Jun;49(6):1126–30.
Teunis 2015	184	Teunis T, Mulder F, Nota SP, Milne LW, Dyer GSM, Ring D. No Difference in Adverse Events Between Surgically Treated Reduced and Unreduced Distal Radius Fractures. J Orthop Trauma. 2015 Nov;29(11):521–5.
Thakur 2021	185	Thakur S.K., Choudhary S.K., Mal J.J.B., Hiremath R.N. Primary bone grafting and k-wire fixation: A preferable option to treat acute unstable scaphoid fracture. Asian J Pharm Clin Res. 2021;14(9):53–6.
Truntzer 2018	186	Truntzer J, Mertz K, Eppler S, Gardner M, Kamal R, Li K. Complication rates by surgeon type after open treatment of distal radius fractures. Eur J Orthop Surg Traumatol. 2018 Dec;28(8):1543–7.
Tyllianakis 2010	187	Tyllianakis M, Mylonas S, Saridis A, Kallivokas A, Kouzelis A, Megas P. Treatment of unstable distal radius fractures with Ilizarov circular, nonbridging external fixator. Injury. 2010 Mar;41(3):306–11.

Van Leeuwen 2016	188	van Leeuwen WF, van Hoorn BTJA, Chen N, Ring D. Kirschner wire pin site infection in hand and wrist fractures: incidence rate and risk factors. J Hand Surg Eur Vol. 2016 Nov;41(9):990–4.
Vasudevan 2017	189	Vasudevan PN, Lohith BM. Management of distal radius fractures - A new concept of closed reduction and standardised percutaneous 5-pin fixation. Trauma. 2018 Apr;20(2):121–30.
Wang W 2021	190	Wang W., Zeng M., Yang J., Wang L., Xie J., Hu Y. Clinical efficacy of closed reduction and percutaneous parallel K-wire interlocking fixation of first metacarpal base fracture. J Orthop Surg. 2021;16(1):454.
Wang L 2021	191	Wang L., Liu H., Ma T., Wu X., Zhang L. Reconstruction of Soft Tissue Defects in the Hand with a Free Anterolateral Thigh Deep Fascia Flap. Orthop Surg. 2021;13(3):758–67.
Wang H 2021	192	Wang H, Yang XX, Huo YX, Qin HY, Wang W, Wang B, et al. [Clinical effects of ulnar artery perforator chain flaps in repairing wounds on distal forearm or wrist with vascular anastomosis]. Zhonghua Shao Shang Za Zhi Zhonghua Shaoshang Zazhi Chin J Burns. 2021;37(7):635–9.
Wang 2022	193	Wang J., Huang Z., Cueva Jumbo J.C., Sha K. Long-term follow-up of one-stage artificial dermis reconstruction surgery for fingertip defects with exposed phalanx. Hand Surg Rehabil. 2022;41(3):353–61.
Wei 2021	194	Wei C., Gu A., Almeida N.D., Bestourous D., Quan T., Fassihi S.C., et al. Operation time effect on rates of perioperative complications after operative treatment of distal radius fractures. J Orthop. 2021;24((Wei, Almeida, Bestourous, Quan, Recarey, Malahias) George Washington School of Medicine and Health Sciences, 2300 Eye St NW, Washington, DC 20037, United States):82–5.
Weinand 2014	195	Weinand C, Demir E, Lefering R, Juon B, Voegelin E. A comparison of complications in 400 patients after native nail versus silicone nail splints for fingernail splinting after injuries. World J Surg. 2014 Oct;38(10):2574–9.
Werber 2003	196	Werber KD, Brauer RB, Raeder F, Weiss S. External fixation of distal radial fractures: Four compared with five pins a randomized prospective study. J Bone Jt Surg - Ser A. 2003 Apr;85(4):660–6.
Whittaker 2005	197	Whittaker JP, Nancarrow JD, Sterne GD. The role of antibiotic prophylaxis in clean incised hand injuries: A prospective randomized placebo controlled double blind trial. J Hand Surg. 2005 May;30(2):162–7.
Yim 2004	198	Yim KK, Wei FC, Lin CH. A comparison between primary and secondary toe-to-hand transplantation. Plast Reconstr Surg. 2004 Jul;114(1):107–12.
Zettl 2009	199	Zettl RP, Clauberg E, Nast-Kolb D, Ruchholtz S, Kühne CA. [Volar locking compression plating versus dorsal plating for fractures of the distal radius: a prospective, randomized study]. Unfallchirurg. 2009 Aug;112(8):712–8.
Zhang 2010	200	Zhang X, Meng H, Shao X, Wen S, Zhu H, Mi X. Pull-out wire fixation for acute mallet finger fractures with k-wire stabilization of the distal interphalangeal joint. J Hand Surg. 2010 Nov;35(11):1864–9.
Zhou 2008	201	Zhou F, Shen B, Wang R, Fan S, Hu W. [Clinical contrast of percutaneous pinning with plaster splint and open reduction and pulling out wire in the treatment of mallet fingers]. Zhongguo Xiu Fu Chong Jian Wai Ke Za Zhi Zhongguo Xiufu Chongjian Waike Zazhi Chin J Reparative Reconstr Surg. 2008 Dec;22(12):1451–4.

Supplementary Table 1 - Characteristics of RCTs

**Author Year**	**Site**	**Type**	**Intervention**	**Study Type**	**Study Design**	**Infection Definition**	**Risk of Bias**	**SSI**	**Total**
Grossman 1981	Hand	Open	STR	Prospective	RCT	Unknown	Moderate	3	265
Peacock 1988	Hand	Mixed	Mixed	Prospective	RCT	Classification	High	1	87
Horton 2003	Hand	Closed	K-wire	Prospective	RCT	Clinical	High	6	15
Werber 2003	Wrist	Closed	Ex-Fix	Prospective	RCT	Clinical	Moderate	6	50
Hargreaves 2004	Wrist	Closed	K-wire	Prospective	RCT	Classification	High	12	56
Azzopardi 2005	Wrist	Closed	K-wire	Prospective	RCT	Clinical	High	1	30
Whittaker 2005	Mixed	Open	STR	Prospective	RCT	Classification	Moderate	17	157
Egol 2006	Wrist	Mixed	Ex-Fix	Prospective	RCT	Classification	High	21	118
Rafique 2006	Hand	Open	K-wire	Prospective	RCT	Clinical	High	12	60
Zettl 2009	Wrist	Closed	ORIF	Prospective	RCT	Clinical	HIgh	1	120
Aydin 2010	Hand	Mixed	Mixed	Prospective	RCT	Classification	High	24	818
Foldhazy 2010	Wrist	Closed	Mixed	Prospective	RCT	Clinical	High	4	22
Hove 2010	Wrist	Closed	Ex-Fix	Prospective	RCT	Clinical	Moderate	19	70
Kivi 2011	Wrist	Closed	K-wire	Prospective	RCT	Clinical	High	15	99
Berwald 2014	Hand	Open	STR	Prospective	RCT	Clinical	High	1	73
Costa 2014	Wrist	Closed	Mixed	Prospective	RCT	Classification	Low	32	461
Chung 2019	Wrist	Closed	Mixed	Prospective	RCT	Clinical	Moderate	30	183
Dias 2020	Wrist	Closed	K-wire	Prospective	RCT	Classification	Low	2	203
Lawson 2021	Wrist	Closed	ORIF	Prospective	RCT	Clinical	Low	0	80
Maradei-Pereira 2021	Wrist	Closed	K-wire	Prospective	RCT	Classification	Moderate	14	220
Mishra 2021	Wrist	Closed	Mixed	Prospective	RCT	Unknown	High	3	62
Noor 2021	Hand	Closed	Mixed	Prospective	RCT	Unknown	High	7	56
Costa 2022	Wrist	Closed	K-wire	Prospective	RCT	Classification	Low	4	245
Khan 2022	Hand	Mixed	K-wire	Prospective	RCT	Classification	High	21	122
Lee 2022	Mixed	Open	STR	Prospective	RCT	Clinical	High	2	25
Lundqvist 2022	Wrist	Closed	ORIF	Prospective	RCT	Clinical	High	3	147
Supichyangur 2022	Hand	Closed	Mixed	Prospective	RCT	Clinical	High	1	23

STR = soft tissue reconstruction, Ex-Fix = external fixation, ORIF = open reduction internal fixation, RCT = randomised control trial

Supplementary Table 2 - Characteristics of Cohort Studies

Author Year	Site	Type	Intervention	Study Type	Study Design	Infection Definition	Risk of Bias	SSI	Total
Suprock 1990	Hand	Open	Mixed	Prospective	Cohort	Clinical	High	8	91
Platt 1995	Hand	Open	Mixed	Prospective	Cohort	Clinical	Moderate	13	124
Shetty 1997	Hand	Open	STR	Retrospective	Cohort	Clinical	High	0	102
Stone 1998	Hand	Open	STR	Retrospective	Cohort	Clinical	High	3	140
Goslings 1999	Wrist	Mixed	Ex-Fix	Prospective	Cohort	Clinical	Low	9	39
Joosten 1999	Wrist	Mixed	Ex-Fix	Prospective	Cohort	Clinical	High	20	174
Lassner 2001	Hand	Mixed	STR	Retrospective	Cohort	Unknown	Moderate	3	48
Lallemand 2002	Hand	Mixed	ORIF	Retrospective	Cohort	Unknown	High	1	43
Yim 2004	Hand	Open	STR	Retrospective	Cohort	Clinical	Low	2	26
Al-Qattan 2005	Hand	Mixed	STR	Retrospective	Cohort	Clinical	Moderate	0	73
Ahmad 2006	Hand	Mixed	Mixed	Retrospective	Cohort	Clinical	High	1	38
Benson 2006	Hand	Open	Mixed	Retrospective	Cohort	Clinical	Moderate	7	34
Fu 2006	Wrist	Mixed	Ex-Fix	Prospective	Cohort	Clinical	Low	1	98
Kurzen 2006	Hand	Mixed	ORIF	Retrospective	Cohort	Clinical	Moderate	3	54
McCallister 2006	Hand	Closed	STR	Prospective	Cohort	Clinical	Low	2	26
Ochman 2006	Wrist	Closed	Ex-Fix	Retrospective	Cohort	Unknown	High	7	67
Zhou 2008	Hand	Closed	Mixed	Retrospective	Cohort	Clinical	High	3	72
Egol 2010	Wrist	Mixed	Mixed	Retrospective	Cohort	Unknown	High	0	44
Gereli 2010	Wrist	Mixed	Mixed	Retrospective	Cohort	Clinical	High	3	30
Lattman 2011	Wrist	Closed	Mixed	Prospective	Cohort	Unknown	High	1	245
Nygaard 2011	Hand	Mixed	Mixed	Retrospective	Cohort	Clinical	High	24	51
Al-Qattan 2012	Hand	Open	Mixed	Retrospective	Cohort	Clinical	Moderate	2	36
Faruqui 2012	Hand	Closed	K-wire	Retrospective	Cohort	Clinical	High	1	50
Mehdinasab 2012	Hand	Open	STR	Prospective	Cohort	Unknown	High	0	32
Ghosh 2013	Hand	Open	K-wire	Prospective	Cohort	Clinical	High	4	30
Kim 2013	Wrist	Mixed	ORIF	Retrospective	Cohort	Clinical	Moderate	3	60
Frueh 2014	Hand	Open	STR	Retrospective	Cohort	Clinical	High	1	191
Lutz 2014	Wrist	Mixed	K-wire	Prospective	Cohort	Unknown	Moderate	16	129

Weinand 2014	Hand	Open	STR	Retrospective	Cohort	Clinical	Moderate	67	401
Lauder 2015	Wrist	Mixed	ORIF	Prospective	Cohort	Unknown	High	0	18
Miao 2015	Hand	Closed	Ex-Fix	Prospective	Cohort	Unknown	High	6	41
Teunis 2015	Wrist	Closed	ORIF	Retrospective	Cohort	Clinical	Low	11	1511
Van Leeuwen 2016	Mixed	Open	K-wire	Retrospective	Cohort	Clinical	Low	85	1213
Basat 2017	Hand	Open	Mixed	Retrospective	Cohort	Clinical	Moderate	9	61
Donnally 2017	Mixed	Mixed	Mixed	Retrospective	Cohort	Clinical	High	6	41
Jose 2017	Wrist	Closed	ORIF	Prospective	Cohort	Clinical	High	1	53
Ridley 2017	Mixed	Open	K-wire	Retrospective	Cohort	Clinical	Low	99	695
Nguyen 2018	Hand	Open	STR	Retrospective	Cohort	Clinical	High	0	30
Terndrup 2018	Hand	Closed	K-wire	Retrospective	Cohort	Clinical	Low	21	444
Truntzer 2018	Wrist	Closed	ORIF	Retrospective	Cohort	Clinical	Low	154	7890
Lutsky 2019	Mixed	Mixed	K-wire	Prospective	Cohort	Classification	Low	17	141
Minhas 2019	Hand	Mixed	Mixed	Retrospective	Cohort	Clinical	Low	31	3506
Ornelli 2019	Hand	Mixed	STR	Retrospective	Cohort	Unknown	High	0	12
Saied 2019	Hand	Closed	K-wire	Prospective	Cohort	Clinical	High	2	61
Tareen 2019	Wrist	Open	Mixed	Retrospective	Cohort	Clinical	High	14	92
Davies 2020	Hand	Open	Mixed	Prospective	Cohort	Clinical	Low	41	983
Henry 2020	Wrist	Open	ORIF	Retrospective	Cohort	Unknown	High	0	24
Lee 2020	Wrist	Mixed	ORIF	Retrospective	Cohort	Clinical	Low	85	1921
Liu Y 2020	Wrist	Mixed	Ex-Fix	Retrospective	Cohort	Unknown	High	15	207
Al-Shahwanii 2021	Wrist	Closed	Mixed	Prospective	Cohort	Clinical	High	5	26
Baldwin 2021	Mixed	Mixed	Mixed	Retrospective	Cohort	Classification	Low	20	556
Baumgartner 2021	Hand	Closed	ORIF	Retrospective	Cohort	Clinical	Moderate	0	79
Bennur 2021	Wrist	Closed	Mixed	Prospective	Cohort	Clinical	High	6	80
Chul-Ho 2021	Hand	Closed	K-wire	Retrospective	Cohort	Unknown	Moderate	1	25
Feldman 2021	Hand	Mixed	Mixed	Retrospective	Cohort	Clinical	Moderate	7	107
Galivanche 2021	Wrist	Mixed	ORIF	Retrospective	Cohort	Clinical	Low	383	16990
Gumussuyu 2021	Hand	Closed	K-wire	Retrospective	Cohort	Clinical	Moderate	1	21
Johnson 2021	Mixed	Open	Mixed	Retrospective	Cohort	Clinical	High	2	89

STR = soft tissue reconstruction, Ex-Fix = external fixation, ORIF = open reduction internal fixation

Jokhio 2021	Wrist	Closed	ORIF	Prospective	Cohort	Clinical	High	1	200
Langridge 2021	Hand	Closed	Mixed	Retrospective	Cohort	Clinical	Moderate	3	49
Levy 2021	Mixed	Mixed	K-wire	Retrospective	Cohort	Clinical	Moderate	0	90
Liu 2021	Hand	Open	STR	Retrospective	Cohort	Clinical	Moderate	5	24
Liu Q 2021	Hand	Open	STR	Retrospective	Cohort	Unknown	High	5	24
Meaike 2021	Wrist	Closed	ORIF	Retrospective	Cohort	Unknown	High	0	115
Raza 2021	Wrist	Closed	K-wire	Retrospective	Cohort	Unknown	High	5	50
Shakoor 2021	Hand	Closed	ORIF	Prospective	Cohort	Unknown	High	3	16
Sim 2021	Mixed	Open	Mixed	Retrospective	Cohort	Clinical	Moderate	3	232
Starr 2021	Hand	Open	Mixed	Retrospective	Cohort	Clinical	Low	2	27
Thakur 2021	Wrist	Closed	ORIF	Prospective	Cohort	Unknown	High	0	21
Wang 2021	Hand	Mixed	STR	Retrospective	Cohort	Unknown	Moderate	0	6
Wang 2021	Wrist	Open	STR	Retrospective	Cohort	Clinical	High	0	11
Wei 2021	Wrist	Mixed	Mixed	Retrospective	Cohort	Classification	Moderate	57	39275
Ahmad 2022	Wrist	Closed	Mixed	Retrospective	Cohort	Clinical	Moderate	6	190
Constantine 2022	Wrist	Mixed	Mixed	Retrospective	Cohort	Clinical	Moderate	781	87169
Hery 2022	Hand	Open	Mixed	Prospective	Cohort	Classification	Moderate	5	405
Kadhum 2022	Mixed	Open	STR	Retrospective	Cohort	Clinical	High	7	151
Li 2022	Hand	Mixed	Mixed	Retrospective	Cohort	Clinical	High	1	70
Mahmood 2022	Wrist	Closed	ORIF	Retrospective	Cohort	Clinical	Moderate	456	132650
Michael 2022	Wrist	Mixed	Ex-Fix	Prospective	Cohort	Clinical	Moderate	3	32
Rao 2022	Hand	Open	STR	Retrospective	Cohort	Clinical	Moderate	0	78
Skochdopole 2022	Wrist	Closed	ORIF	Retrospective	Cohort	Unknown	Moderate	13	5894

Supplementary Table 3 - Characteristics of Case Control Studies

Author Year	Site	Type	Intervention	Study Type	Study Design	Infection Definition	Risk of Bias	SSI	Total
Bednar 2004	Wrist	Closed	Ex-Fix	Retrospective	Case control	Clinical	High	2	12
Dwyer 2017	Wrist	Closed	Mixed	Retrospective	Case control	Unknown	High	6	25

Ex-Fix = external fixation

Supplementary Table 4 - Characteristics of Case Series

Author Year	Site	Type	Intervention	Study Type	Study Design	Infection Definition	Risk of Bias	SSI	Total
Conolly 1973	Hand	Mixed	Mixed	Retrospective	Case series	Clinical	High	18	260
Marshall 1978	Hand	Open	STR	Retrospective	Case series	Unknown	High	0	7
Grundberg 1981	Hand	Mixed	ORIF	Retrospective	Case series	Unknown	High	0	21
Adkison 1982	Wrist	Mixed	Mixed	Retrospective	Case series	Clinical	High	1	54
Smith 1988	Wrist	Closed	ORIF	Retrospective	Case series	Clinical	Moderate	0	16
Swanson 1991	Hand	Closed	Mixed	Retrospective	Case series	Clinical	Moderate	7	117
Botte 1992	Mixed	Closed	K-wire	Retrospective	Case series	Clinical	Moderate	10	137
Duncan 1993	Hand	Closed	Mixed	Retrospective	Case series	Clinical	Moderate	6	75
Eglseder 1993	Wrist	Mixed	Ex-Fix	Retrospective	Case series	Clinical	High	0	19
Starker 1995	Hand	Open	K-wire	Prospective	Case series	Clinical	High	0	68
Hove 1997	Wrist	Closed	Ex-Fix	Retrospective	Case series	Unknown	High	1	29
Lopes 1997	Wrist	Closed	ORIF	Retrospective	Case series	Unknown	High	1	19
Ring 1997	Wrist	Closed	ORIF	Prospective	Case series	Clinical	Moderate	0	22
Page 1998	Hand	Open	ORIF	Retrospective	Case series	Clinical	Moderate	3	82
Bauze 1999	Hand	Closed	ORIF	Retrospective	Case series	Clinical	Moderate	2	10
Ignacio 1999	Wrist	Mixed	Ex-Fix	Prospective	Case series	Unknown	High	5	25
Stahl 2001	Mixed	Open	K-wire	Retrospective	Case series	Clinical	Moderate	13	236
Bhat 2002	Hand	Open	K-wire	Retrospective	Case series	Clinical	High	11	120
Akmaz 2003	Wrist	Mixed	Ex-Fix	Retrospective	Case series	Clinical	High	4	25
Lee 2003	Wrist	Closed	ORIF	Retrospective	Case series	Clinical	Moderate	1	22
Anderson 2004	Wrist	Mixed	Ex-Fix	Retrospective	Case series	Clinical	Moderate	9	24
Gradl 2005	Wrist	Closed	Ex-Fix	Prospective	Case series	Clinical	Low	3	25
Kömürcü 2005	Wrist	Mixed	Ex-Fix	Retrospective	Case series	Clinical	Moderate	4	24
Bach 2006	Hand	Open	ORIF	Retrospective	Case series	Clinical	Moderate	0	10
Rocchi 2006	Hand	Closed	K-wire	Retrospective	Case series	Clinical	Moderate	1	48
Saint-Cyr 2006	Hand	Open	ORIF	Retrospective	Case series	Clinical	Moderate	0	7
Ali 2007	Hand	Mixed	Mixed	Prospective	Case series	Clinical	High	5	94
Dennison 2007	Wrist	Closed	ORIF	Retrospective	Case series	Clinical	Moderate	0	5

Dumont 2007	Hand	Closed	ORIF	Retrospective	Case series	Clinical	High	0	12
Al-Qattan 2008	Hand	Closed	ORIF	Prospective	Case series	Clinical	Moderate	0	15
Henry 2008	Hand	Mixed	STR	Retrospective	Case series	Clinical	Moderate	7	108
Nazerani 2009	Hand	Open	STR	Retrospective	Case series	Unknown	Moderate	0	24
Anakwe 2010	Wrist	Closed	ORIF	Prospective	Case series	Clinical	Moderate	0	21
Tyllianakis 2010	Wrist	Open	Ex-Fix	Retrospective	Case series	Clinical	Moderate	7	20
Zhang 2010	Hand	Closed	K-wire	Prospective	Case series	Clinical	Low	0	65
Al-Qattan 2011	Hand	Closed	K-wire	Retrospective	Case series	Clinical	Low	4	35
Campochiaro 2011	Wrist	Closed	Mixed	Retrospective	Case series	Clinical	Moderate	0	77
Capo 2011	Hand	Open	Mixed	Retrospective	Case series	Clinical	Moderate	2	102
Chafik 2011	Hand	Closed	K-wire	Prospective	Case series	Clinical	High	5	24
Nazerani 2011	Hand	Mixed	STR	Retrospective	Case series	Unknown	High	4	64
Szalay 2011	Hand	Closed	ORIF	Retrospective	Case series	Clinical	Low	0	77
Cheah 2012	Hand	Closed	ORIF	Retrospective	Case series	Clinical	Moderate	0	13
Marsland 2012	Hand	Closed	Ex-Fix	Retrospective	Case series	Clinical	High	3	8
Reissner 2012	Hand	Closed	K-wire	Retrospective	Case series	Clinical	Low	5	32
An 2013	Hand	Closed	K-wire	Retrospective	Case series	Clinical	High	0	27
Hussain 2013	Hand	Open	STR	Retrospective	Case series	Unknown	High	0	113
Mattiassich 2013	Hand	Closed	K-wire	Retrospective	Case series	Unknown	Moderate	4	26
Tarallo 2013	Wrist	Closed	ORIF	Retrospective	Case series	Clinical	Moderate	1	303
Aigner 2014	Wrist	Mixed	Mixed	Retrospective	Case series	Clinical	Moderate	6	721
Boussakri 2014	Hand	Closed	K-wire	Retrospective	Case series	Clinical	High	3	28
Rajappa 2014	Mixed	Open	STR	Prospective	Case series	Clinical	Moderate	1	26
Brei-Thoma 2015	Hand	Closed	ORIF	Retrospective	Case series	Clinical	Moderate	0	32
de Haseth 2015	Hand	Closed	K-wire	Retrospective	Case series	Clinical	Moderate	1	9
Erken 2015	Hand	Open	STR	Retrospective	Case series	Unknown	Moderate	0	12
Kilic 2015	Hand	Open	STR	Retrospective	Case series	Clinical	High	21	67
Matullo 2015	Wrist	Mixed	ORIF	Retrospective	Case series	Unknown	Moderate	1	21
Pelissier 2015	Hand	Closed	K-wire	Retrospective	Case series	Clinical	Moderate	4	86
Fan 2016	Wrist	Mixed	ORIF	Retrospective	Case series	Unknown	High	0	12

STR = soft tissue reconstruction, Ex-Fix = external fixation, ORIF = open reduction internal fixation

Li C 2016	Hand	Closed	ORIF	Prospective	Case series	Unknown	Low	0	66
Li S 2016	Hand	Mixed	K-wire	Retrospective	Case series	Unknown	Moderate	0	16
Parmaksizoglu 2016	Hand	Open	STR	Retrospective	Case series	Clinical	Moderate	0	3
Prunieres 2016	Hand	Open	K-wire	Prospective	Case series	Clinical	High	0	12
Rabarin 2016	Hand	Open	STR	Retrospective	Case series	Clinical	Moderate	0	28
Johandi 2017	Wrist	Mixed	STR	Retrospective	Case series	Unknown	Moderate	1	12
Vasudevan 2017	Wrist	Closed	K-wire	Retrospective	Case series	Clinical	Moderate	52	458
Ficke 2018	Wrist	Mixed	ORIF	Retrospective	Case series	Clinical	Moderate	5	47
Huang 2018	Wrist	Mixed	ORIF	Retrospective	Case series	Unknown	Low	0	24
Guerro 2019	Hand	Mixed	ORIF	Retrospective	Case series	Clinical	Low	2	23
Kucukguven 2019	Hand	Open	STR	Prospective	Case series	Unknown	Low	4	23
Singh 2019	Hand	Closed	ORIF	Retrospective	Case series	Clinical	Moderate	1	11
Abouelela 2020	Hand	Closed	K-wire	Retrospective	Case series	Clinical	Moderate	1	33
Afshar 2020	Hand	Closed	ORIF	Prospective	Case series	Clinical	Low	0	21
Cheng 2020	Wrist	Closed	K-wire	Retrospective	Case series	Clinical	Low	2	17
Kural 2020	Hand	Closed	Mixed	Retrospective	Case series	Clinical	High	0	11
Pan 2020	Hand	Open	STR	Prospective	Case series	Clinical	High	0	17
Tarallo 2020	Wrist	Closed	ORIF	Retrospective	Case series	Clinical	Low	1	110
Abulsoud 2021	Hand	Closed	K-wire	Prospective	Case series	Unknown	Moderate	0	23
Beeres 2021	Wrist	Mixed	ORIF	Retrospective	Case series	Clinical	High	0	12
Camacho 2021	Hand	Mixed	ORIF	Retrospective	Case series	Unknown	High	0	45
Martins 2021	Hand	Open	STR	Retrospective	Case series	Unknown	High	0	37
Meena 2021	Wrist	Mixed	ORIF	Retrospective	Case series	Clinical	High	2	25
Solari 2021	Hand	Closed	K-wire	Retrospective	Case series	Clinical	Low	9	200
Wang 2021	Hand	Closed	K-wire	Retrospective	Case series	Unknown	Low	0	20
Wang 2021	Hand	Closed	K-wire	Retrospective	Cohort	Unknown	High	0	20
Arora 2022	Wrist	Closed	ORIF	Retrospective	Case series	Clinical	Moderate	1	28
Choubey 2022	Wrist	Closed	ORIF	Prospective	Case series	Clinical	Moderate	1	28
Ibrahim 2022	Mixed	Open	Mixed	Retrospective	Case series	Clinical	High	0	17
Joo 2022	Wrist	Open	ORIF	Retrospective	Case series	Clinical	High	2	24

Liechti 2022	Wrist	Closed	ORIF	Prospective	Case series	Clinical	Low	0	28
Naguib 2022	Hand	Closed	Ex-Fix	Prospective	Case series	Clinical	Low	1	20
Wang 2022	Hand	Open	STR	Retrospective	Case series	Clinical	Moderate	0	24
